# Deep learning of MRI contrast enhancement for mapping cerebral blood volume from single-modal non-contrast scans of aging and Alzheimer's disease brains

**DOI:** 10.3389/fnagi.2022.923673

**Published:** 2022-08-11

**Authors:** Chen Liu, Nanyan Zhu, Haoran Sun, Junhao Zhang, Xinyang Feng, Sabrina Gjerswold-Selleck, Dipika Sikka, Xuemin Zhu, Xueqing Liu, Tal Nuriel, Hong-Jian Wei, Cheng-Chia Wu, J. Thomas Vaughan, Andrew F. Laine, Frank A. Provenzano, Scott A. Small, Jia Guo

**Affiliations:** ^1^Department of Electrical Engineering, Columbia University, New York, NY, United States; ^2^Department of Biological Sciences, Columbia University, New York, NY, United States; ^3^Department of Biomedical Engineering, Columbia University, New York, NY, United States; ^4^Department of Pathology and Cell Biology, Columbia University, New York, NY, United States; ^5^Department of Radiation Oncology, Columbia University, New York, NY, United States; ^6^Department of Neurology, Columbia University, New York, NY, United States; ^7^Department of Psychiatry, Columbia University, New York, NY, United States; ^8^Taub Institute for Research on Alzheimer's Disease and the Aging Brain, Columbia University, New York, NY, United States; ^9^The Mortimer B. Zuckerman Mind Brain Behavior Institute, Columbia University, New York, NY, United States

**Keywords:** aging, CBV, deep-learning, gadolinium, MRI, Alzheimer's disease

## Abstract

While MRI contrast agents such as those based on Gadolinium are needed for high-resolution mapping of brain metabolism, these contrast agents require intravenous administration, and there are rising concerns over their safety and invasiveness. Furthermore, non-contrast MRI scans are more commonly performed than those with contrast agents and are readily available for analysis in public databases such as the Alzheimer's Disease Neuroimaging Initiative (ADNI). In this article, we hypothesize that a deep learning model, trained using quantitative steady-state contrast-enhanced structural MRI datasets, in mice and humans, can generate contrast-equivalent information from a single non-contrast MRI scan. The model was first trained, optimized, and validated in mice, and was then transferred and adapted to humans. We observe that the model can substitute for Gadolinium-based contrast agents in approximating cerebral blood volume, a quantitative representation of brain activity, at sub-millimeter granularity. Furthermore, we validate the use of our deep-learned prediction maps to identify functional abnormalities in the aging brain using locally obtained MRI scans, and in the brain of patients with Alzheimer's disease using publicly available MRI scans from ADNI. Since it is derived from a commonly-acquired MRI protocol, this framework has the potential for broad clinical utility and can also be applied retrospectively to research scans across a host of neurological/functional diseases.

## Introduction

Gadolinium-based contrast agents (GBCAs) are intravenously administered with Magnetic Resonance Imaging (MRI), and they are most widely known for their utility in mapping, enhancing, and detecting structural brain lesions such as those found in cancer, infections, or bleeding (Borges et al., 2012; Lohrke et al., 2016; Shen and Duong, 2016). Another utility of GBCAs, far less popular until recent years, is to identify functional abnormalities, such as those that alter brain metabolism in aging, neuropsychiatric disorders, and neurodegenerative diseases (Belliveau, 1991; Lewandowski et al., 2013; Schobel et al., 2013; Khan et al., 2014). For these functional lesions, GBCAs can be used to generate high-resolution cerebral blood volume (CBV) maps, thereby deriving a quantitative correlate of metabolic dysfunction that is otherwise invisible to MRI without contrast.

However, recent studies have begun to call the safety of GBCAs into question (Quattrocchi and van der Molen, 2017; Ramalho et al., 2017; Guo et al., 2018; Dillman and Davenport, 2020). GBCAs contain gadolinium, a heavy metal, and are injected into a vein to improve the visualization of internal organs, blood vessels, and tissues during an MRI. After being administered, GBCAs are mostly cleared out from the body through the kidneys. However, trace amounts of gadolinium may stay in patients' bodies, including the brain, for months to years after receiving GBCAs. GBCAs may also increase the risk of nephrogenic systemic fibrosis, a rare but serious disease, in people with severe kidney failure. Even if these concerns are addressed, GBCA administration requires intravenous access, a requirement that places risks on patients and healthcare practitioners, as well as limiting its application in cases when contraindicated (Guo et al., 2018). Thus, there is a need to find a “GBCA substitute”, one that can generate GBCA-equivalent information from a non-contrast MRI scan.

Apart from other alternatives, one solution may lie in the non-contrast MRI scans themselves. The main purpose of GBCAs is to selectively highlight signals from the blood so that the blood vessels can visually stand out from the surrounding brain tissues. However, though not visually apparent, such blood-tissue contrast is also present even in non-contrast MRI scans. The underlying reason comes from magnetic resonance physics. The intensity of a voxel in a non-contrast MRI scan is determined by the physical properties, namely the proton density (PD), the T1-, T2-, and T2*- relaxation time constants, of the corresponding material within that voxel. Importantly, blood and different brain tissues have distinct T1 relaxation time constants. At 3 Tesla, the average T1 relaxation time constants of the white matter, the gray matter, and the blood are 866.9, 1433.2, and 1984.4 ms, respectively (Hasgall et al., 2022). In addition, a previous study has shown that some dark structures, identified mainly as vessels, are frequently misclassified as the cerebrospinal fluid (CSF) in T2/PD MRI (Dugas-Phocion et al., 2004) and further demonstrated that ignoring vessel contrast when handling partial volume effect can also lead to an over-estimation of the CSF variance in the intensity space. Other studies have also shown that vessels appear darker than brain tissues on T2*-weighted gradient echo magnetic resonance (GRE) images due to shorter T2* relaxation (Small et al., 2000), and this contrast between blood vessels and brain tissues has been named the susceptibility vessel sign (SVS) (Flacke et al., 2000; Rovira et al., 2004). Hence, non-contrast MRI scans are theoretically able to yield patterns of voxel intensities to distinguish blood vessels from surrounding tissues, though such differences are too subtle to reliably detect and quantify with previous analytical or qualitative methods. Nevertheless, according to magnetic resonance physics, at least a fraction of the GBCA-contrast information due to blood vessels is present and partially encoded, in non-contrast structural MRI scans through a non-linear function.

Deep learning, a subset of machine learning, is an established method for approximating non-linear functions using a data-driven approach. A deep learning model should, therefore, be able to learn how to optimally extract key features at a voxel level, by inspecting MRI scans where GBCAs were administered along with their non-contrast counterparts. As such, a growing number of recent studies have begun validating this assumption (Kleesiek et al., 2019; Liu et al., 2019; Li et al., 2021). Among these, one study managed to use deep learning to reduce the GBCA dose (Gong et al., 2018), but not to completely substitute for it. Other studies succeeded in obviating the need for GBCA (Kleesiek et al., 2019; Liu et al., 2019; Li et al., 2021) but these deep learning models require the acquisition of an array of multiple MRI sequences, some of which are not widely or clinically available. Among these studies that succeeded in obviating the need for GBCA, more modern deep learning-based methods are especially well suited for this task and demonstrated favorable performance: for the BayesUNet method (Kleesiek et al., 2019) with a comprehensive multiparametric MRI protocol including pre-contrast T1-weighted, T2-weighted, T2-weighted fluid-attenuated inversion recovery (FLAIR), diffusion-weighted imaging (DWI) and susceptibility-weighted imaging (SWI) sequences as the input for predicting synthetic post-contrast T1-weighted sequence, analysis of the whole brain showed a peak signal-to-noise ratio (PSNR) of 22.967 ± 1.162 and a structural similarity index (SSIM) of 0.872 ± 0.031; for the CGAN method (Preetha et al., 2021) with pre-contrast T1-weighted, T2-weighted and FLAIR sequences as the input, the model reached a median SSIM of 0.818 (95% CI 0.817 - 0.820); and the MMgSN-Net method (Li et al., 2021) with pre-contrast T1-weighted and T2-weighted sequences as the input achieved top-ranked scores in averaged PSNR of 33.17 ± 2.14 and SSIM of 0.887 ± 0.042.

With these issues in mind, we hypothesized that a deep learning model could extract GBCA-equivalent information from a single and commonly-acquired high-resolution MRI scan, by training and optimizing the model using a large and unique GBCA MRI dataset. Previous deep learning studies relied on GBCA datasets generated for radiological purposes, where post-GBCA scans are, by necessity, re-scaled in order to facilitate a radiologist's ability to detect and characterize brain abnormalities. Such re-scaling operations are performed in a case-by-case manner without a universal scaling factor, thus increasing the intersubject variability across a dataset.

Through our previous study in mapping functional brain lesions that localize to specific regions of the hippocampal formation, we have extensively used GBCAs to generate quantitative, high-resolution CBV maps (Small et al., 2011; Pavlopoulos et al., 2013; Schobel et al., 2013; Brickman et al., 2014; Khan et al., 2014; Provenzano et al., 2020). By design, these quantitative maps preserve scaling with respect to the post-GBCA image. While not the original intent, we have accrued a large-scale dataset with reduced inter and intrasubject variability, which we predicted would benefit the training of our model. In parallel to generating a large-scale and quantitative GBCA dataset in humans, we have also accumulated a similar MRI dataset in mice (Moreno et al., 2006; Khan et al., 2014). Again, the original intent was to validate patterns of hippocampal dysfunction observed across disease states; however, because these animal study subjects were siblings with identical genetic backgrounds, this dataset is likely to contain less variability than possible in humans.

**Figure 1:**
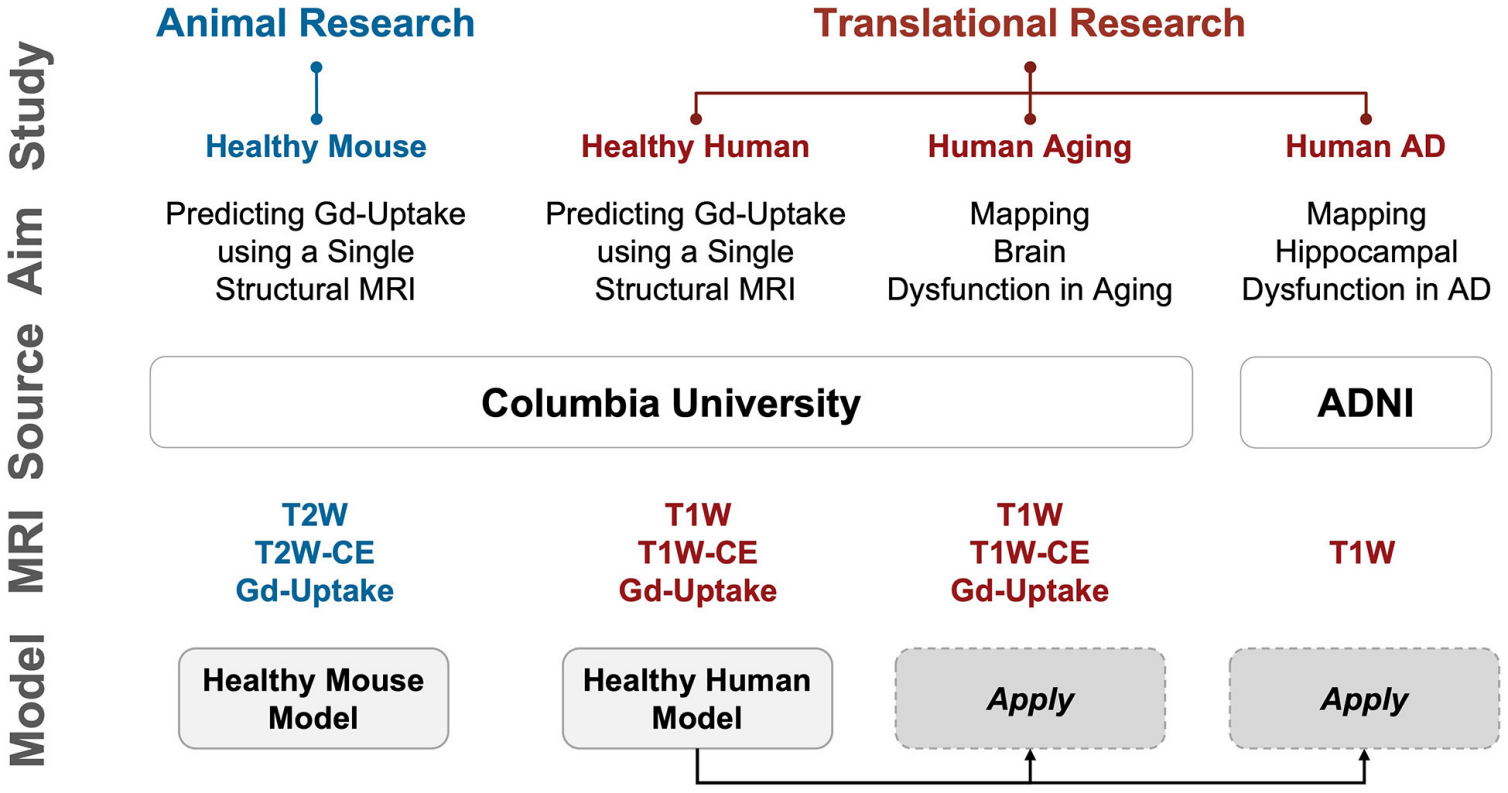
Overview of the studies conducted. We first performed proof-of-concept studies in mice to validate our hypothesis that deep learning can extract information equivalent to Gadolinium-based contrast agent (GBCA) contrast enhancement from a single-modal non-contrast MRI scan, and then conducted extensive analyses in humans to scrutinize the capability of this proposed approach. Study: A study conducted; Aim: The purpose of the study; Source: where the imaging data come from; MRI: modality/type of data used in the study; Model: specific DeepContrast model used in the study. AD: Alzheimer's disease; ADNI: Alzheimer's Disease Neuroimaging Initiative dataset; CBV: cerebral blood volume; Gd-Uptake: GBCA contrast uptake maps; T2W: T2-weighted scans; T2W-CE: T2-weighted contrast-enhanced scans; T1W: T1-weighted scans; T1W-CE: T1-weighted contrast-enhanced scans.

In this study, we exploited this distinct cross-species and quantitative GBCA dataset. Beginning with mice to prove the concept, we first designed, optimized, and trained a deep learning model to synthesize GBCA enhancement in the mouse brain from the T2-weighted structural MRI. We further adapted the proposed deep learning model to the human dataset and validated that it can also indicate GBCA enhancement in the human brain from the T1-weighted MRI. The deep learning model will be referred to herein as “DeepContrast”. We then utilized this DeepContrast technique to study brain aging and Alzheimer's disease, applying it to both in-house datasets from an aging study, as well as to the publicly available ADNI dataset from Alzheimer's patients and age-matched controls. The studies conducted are outlined in Figure 1. Our results demonstrate that trained deep learning contrast enhancement models can successfully identify and localize brain functional changes that occur through aging and Alzheimer's disease previously only identifiable with GBCA methods.

## Materials and methods

As an overview, we conducted 4 sets of studies, as summarized in Figure 1: Healthy Mouse, Healthy Human, Human Aging, and Human AD. In the first two studies, we qualitatively and quantitatively assessed the ability of our proposed DeepContrast model to predict the GBCA enhancement in healthy mice and human brains. In the latter two, we first conducted pilot studies to validate whether the DeepContrast-synthesized CBV maps exhibited the same patterns observed from real CBV data in prior research. Then we performed utility studies to demonstrate the potential use cases. Due to the inherent differences between the Aging and AD studies (presence vs. absence of ground truth, continuous vs. categorical, etc.), the utility studies were designed differently.

In this section, we will describe the data source, data preparation, and detailed analyses performed.

### Animal subjects and human participants

#### Healthy mouse

We used 49 healthy adult C576J/BL male mice (12–14 months old).

#### Healthy human

We aggregated the healthy human MRI data from previous acquisitions at Columbia University. As we mentioned in the Introduction section, these scans were originally acquired for the purposes of mapping functional lesions. This included 598 participants (16–94 years old) with single acquisitions and another 11 participants with baseline and follow-up acquisitions 14 days apart.

#### Human aging

For both the pilot and utility studies, we used scans from 177 participants (20–72 years old) that were cognitively normal. These 177 participants were a subset of the 598 healthy participants. The selection criteria will be described in the following ‘Preprocessing and partitioning' section.

#### Human AD

For the pilot study, we selected 50 cognitively normal (CN) and 50 Alzheimer's disease (AD) participants, each with two back-to-back repeated scans from ADNI (Mueller et al., 2005), resulting in a 100-participant (60–90 years old) dataset. For the utility study, we prepared a larger, 2,580-participant (50–100 years old) dataset from ADNI, with 1290 CN and 1290 AD participants.

### Image acquisition protocols

#### Healthy mouse

We used CBV-fMRI to image male Wildtype (WT) mice used in the healthy mouse study, with the imaging protocol as previously described (Moreno et al., 2006). A Bruker BioSpec 94/30 (field strength, 9.4 T; bore size, 30 cm) horizontal small animal MRI scanner equipped with CryoProbe and software ParaVision 6.0.1 (Bruker BioSpin, Billerica, MA, USA) and a 23-mm 1H circularly polarized transmit/receive capable mouse head volume coil were used for the imaging. Mice were anesthetized using medical air and isoflurane (3% volume for induction, 1.1–1.5% for maintenance at 1 liter/min air flow, *via* a nose cone). A flowing water heating pad was used to maintain the body temperature at around 37°C. Sterile eye lubricant was applied before each scan. T2-weighted images were acquired before and 36 min after intraperitoneal injections of the GBCA-based contrast agent Gadodiamide (Omniscan; GE Healthcare, Princeton, NJ, USA) at the dosage of 10 mmol/kg. T2-weighted images were acquired with Refocused Echoes (RARE) sequence (repetition time (TR) = 3,500 ms, effective echo time (TE) = 45 ms, rapid acquisition and relaxation enhancement (RARE) factor = 8, voxel size = 450×76×76 μm).

#### Healthy human

The images were acquired under a steady-state CBV-fMRI protocol as previously described (Khan et al., 2014). A gradient echo T1-weighted scan (TR = 6.7 ms, TE = 3.1 ms, field of view (FOV) = 240×240×192 mm, voxel size = 0.9×0.9×0.9 mm) was acquired before a pair of un-scaled T1-weighted images (TR = 7 ms, TE = 3 ms, FOV = 240×240×196 mm, voxel size = 0.68×0.68×3 mm), all using a Philips Achieva 3.0-T MRI scanner. The image resolution used results from a systematic exploration of the scan protocol's parameters. Scans were acquired before and after a bolus injection of a GBCA-based contrast agent (Omniscan, GE Healthcare).

#### Human aging

The data used in the Human Aging study was a subset of the Healthy Human study, and hence the protocols were identical.

#### Human AD

The images included in our studies were acquired using a 3D magnetization prepared rapid gradient echo (MP-RAGE) protocol, yielding near-isotropic images (voxel size ≈ 1×1×1 mm). More details can be found in the official documentation of ADNI (Mueller et al., 2005).

### Preprocessing and partitioning

#### Healthy mouse

In total, 49 WT mice were used in this study. Whole brain T2W MRI scans before (T2W) and 35 mins after (T2W-CE) intraperitoneal injection of Gadodiamide were acquired. The Gd-Uptake ground truth was quantified with the standardized delta-R2, which was derived using the same method as discussed before (Moreno et al., 2006), followed by standardization to the dynamic range of [0, 1]. We used 3D PCNN (Chou et al., 2011) with the manual correction to generate brain masks, which we used as training fields over which the model was optimized and performance metrics were calculated. A train-validation-test ratio of 8:1:1 was applied in the Healthy Mouse Model training.

#### Healthy human

T1-weighted MRI scans were acquired using the protocols as described previously (Brickman et al., 2014; Provenzano et al., 2020), before (T1W) and 4 min after (T1W-CE) a bolus intravenous injection of Gadodiamide. Unlike many other similar studies, during the MRI acquisition for the same session, the receiver gain was intentionally kept constant and the offset was set to zero. As a result, the T1W and T1W-CE scans shared the same scaling and zero shifting, and hence the same voxel intensity between each T1W/T1W-CE pair corresponds to the same relaxation-time property in the magnetic resonance physics context. Each T1W and T1W-CE pair was spatially aligned when provided. For intensity normalization, each T1W scan was compressed to the dynamic range of [0, 1], and the corresponding T1W-CE scan was scaled by the same factor to preserve the voxel intensity correspondence. The Gd-Uptake ground truth was quantified with the steady-state MRI method (Brickman et al., 2014), by subtracting the normalized T1W scans from the respective T1W-CE scans. We generated brain masks using the BET function in FMRIB Software Library (FSL) (Jenkinson et al., 2012), which we used as training fields over which the model was optimized and performance metrics were calculated. We generated tissue label maps using the FAST function in FSL for tissue-of-interest analyses. The train-validation-test split yielded 326 for training, and 93 for validation, while 179 participants were left for the test set.

#### Human aging

The 177-participant cohort used for the aging study was a subset of the 179 participants in the test set of the Healthy Human Model, where 2 participants were dropped due to low segmentation quality as defined through a failure of processing the FreeSurfer (v6.0.0) Parcellation. After normalization to the dynamic range of [0, 1], the T1W scans were directly treated as inputs to the model to generate the Gd-Predicted maps. Synthesized CBV maps were then generated by applying the same normalization method on the Gd-Predicted maps as we would quantify CBV maps.

#### Human AD

For the large-scale utility study, we screened T1W MRI scans and excluded all scans except for 3 Tesla MP-RAGE acquisitions (Supplementary Figure S4b top left). After that, we further performed propensity score matching (PSM) to match the age distribution and eventually resulted in a dataset with 1,290 scans of patients with AD and 1,290 scans of age-matched CN volunteers (Supplementary Figure S4b bottom left). A major challenge was that the appearance and anatomy of the scans used in the AD study notably differ from those used to train the DeepContrast Healthy Human Model. They were acquired under the same field strength (i.e., 3 Tesla), but specific scan parameters such as echo time and repetition time are different between the ADNI protocol and the CBV-fMRI protocol. Additionally, the participants in the AD study are generally older (60–90 years old) and half of them harbor Alzheimer's pathology, thus resulting in a potential mismatch in anatomy. We approached these issues by applying (1) affine registration for T1W MRI data and (2) rigid registration to the unbiased MNI152 template on the raw whole brain data and then (3) minimizing the between-cohort appearance difference using a dynamic histogram warping (DHW) algorithm (Cox et al., 1995) as it was demonstrated to be among the best intensity matching methods in medical imaging (Wagenknecht et al., 2000). Specifically, we calculated the mean normalized-brain-region 2048-bin histogram of each cohort derived a bin-to-bin mapping between the cohorts and applied the mapping to each individual scan in the AD study. In step (4), we minimized the anatomical difference by diffeomorphic registration using the Symmetric Normalization (SyN) algorithm (Avants et al., 2009) prior to applying the DeepContrast model. Finally, we normalized the scans to the dynamic range of [0, 1] and provided them to the model to generate the Gd-Predicted maps. Synthesized CBV maps were then generated by applying the same normalization method as we would quantify synthesized CBV maps and up-sampled to a voxel size of 1×1×1 mm. The data pre-processing is illustrated in Supplementary Figure S4a. The prepared cohort with a total of 2580 T1W scans and 2580 synthesized CBV scans, were randomly assigned to train, validation, and test sets at an 8:1:1 ratio. Randomization was performed on the participant level to prevent data leakage. AD and CN participants were independently randomized to balance the presence of both classes in each set. The data distribution was summarized in the right half of the Supplementary Figure S4b.

For the pilot study, we used a subset containing 50 AD and 50 CN participants each with two back-to-back repeated scans. Compared to the large-scale utility study, the sample size was reduced to accommodate the voxel-based data analysis tool (SPM12) used in the pilot study. Data processing was the same as above.

### DeepContrast model implementation

**Figure 2:**
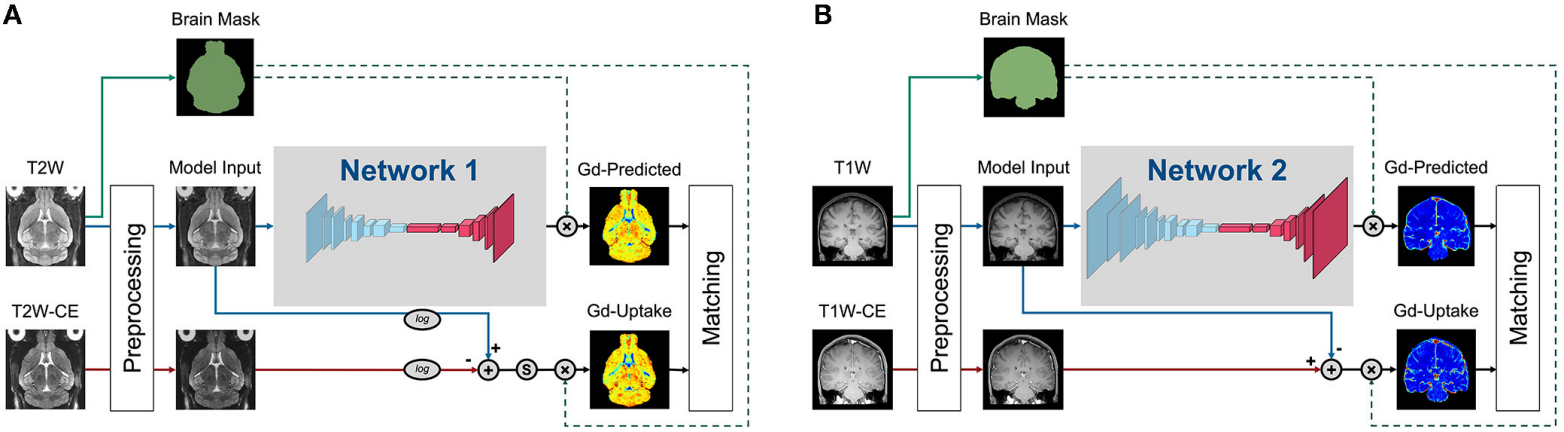
Training strategies of the various DeepContrast models implemented. **(A)** This training strategy applied to the Healthy Mouse Model. Preprocessing includes intensity normalization and brain extraction. Ground truth Gd-Uptake was derived using the standardized delta-R2 equation. Note that there is an additional standardization step that maps the dynamic range of the standardized delta-R2 to the range of [0, 1], before the application of the brain mask. The loss function was calculated between the Gd-Uptake and the predicted version only using the voxels within the brain mask region. **(B)** This training strategy applied to the Healthy Human Model. Preprocessing included intensity normalization and brain extraction. Ground truth Gd-Uptake was derived using the steady-state delta-R1 equation. The loss function was calculated between the Gd-Uptake and the predicted version only using the voxels within the brain mask region.

All model variants developed in our studies, as mentioned in Figure 1, shared the common residual attention U-Net (RAU-Net) architecture (Figure 2). Model inputs were the non-contrast MRI scans, while the outputs were the corresponding predicted GBCA contrast (Gd-Predicted). The inputs and outputs were 2D slices of equal dimension since the MRI scans were acquired under 2D protocols. The slice direction was defined as the axis with the lowest spatial resolution, which was axial for Healthy Mouse scans and coronal for Healthy Human scans.

The RAU-Net is an extension of the arguably most popular deep learning architecture in medical imaging, the U-Net (Ronneberger et al., 2015), with the addition of residual blocks (He et al., 2016) and the attention gates (Vaswani et al., 2017; Oktay et al., 2018). As an example of a convolutional neural network (CNN), the U-Net extracts imaging features by utilizing local convolutions along the entire image or volume. The U-Net consists of multiple encoding layers across which the image dimension shrinks whereas the feature dimension increases so that compact high-level abstractions are generated along the process, and the same number of decoding layers to decipher these abstractions into image space information. The add-on residual blocks simplify the entities to be approximated across each layer and, therefore, enables training of deeper networks, while the attention gates learn to differentially enhance or suppress specific regions in the feature maps so that the downstream outcomes are better represented for targeting objective.

Specifically, the encoding and decoding paths consist of the same number of residual convolution blocks that utilize concatenation, attention mechanisms, and skip connections such that layers feed not only into the next layer but into the layer after the next layer. On the encoding path, each residual block is followed by a max-pooling layer, and the last feature map feeds into a bottleneck layer with 3×3 convolution and batch normalization, connecting the deepest layer to the decoding path with several more blocks alternating one un-pooling layer and one residual block. Skip connections concatenate the output of each dense layer in the encoding path with the respective un-pooled feature map of the same size before feeding it as input to the decoding residual block. The output of the last decoding layer is the input for a 1×1 convolution layer that produces the final Gd-Predicted map.

#### Healthy mouse model

The model (Supplementary Figure S1) used in mouse studies was a 2D RAU-Net that consisted of 5 encoding and decoding layers. The model input was a 2D axial slice of the mouse brain scans. Adam optimizer with a learning rate of 0.001 was used in this study. Our batch size was 3 and the loss function was mean squared error (MSE).

#### Healthy human model

The model (Supplementary Figure S2) used in the healthy human study and further applied to the Aging and AD studies was a 2D RAU-Net that consisted of 6 encoding and decoding layers. The model input was a 2D coronal slice of the human brain scans. SGD optimizer with an adaptive learning rate handle with a 0.1 initial learning rate was used in this study. Our batch size was 4 and a robust adaptive loss function (Barron, 2019) was utilized. The robust adaptive loss function is a generalization of the Cauchy/Lorentzian, Geman-McClure, Welsch/Leclerc, generalized Charbonnier, Charbonnier/pseudo-Huber/L1-L2, and L2 loss functions. By introducing robustness as a continuous parameter, the robust adaptive loss function allows algorithms built around robust loss minimization to be generalized, which improves performance on basic vision tasks like calculating the intensity mapping function in our case.

### Statistical methods

#### Healthy mouse and healthy human

##### Prediction vs. ground truth similarity assessment

Peak signal-to-noise ratio (PSNR), structural similarity index (SSIM) (Wang et al., 2004), Pearson correlation coefficient (P.R), and Spearman correlation coefficient (SR) were used to quantify the performance of all the DeepContrast models. PSNR, Pearson correlation coefficient, and Spearman correlation coefficient were evaluated within the brains or subregions, and SSIM was calculated in the minimum bounding box around the brains or subregions.

#### Human aging

##### Pilot study part 1: Voxel-based analysis on the hippocampal circuit

Voxel-based analysis (**Figures 4B,C**) was performed by first transforming the non-contrast images using a diffeomorphic registration algorithm (Avants et al., 2009) with nearest-neighbor interpolation to an unbiased brain template created from the 177 scans in the Aging study (Avants et al., 2009). The GBCA-predicted maps were generated by the Healthy Human model using the native-space non-contrast T1W scans as the input and were subsequently used to generate synthesized CBV maps by normalization using the mean value among the top 10% brightest voxels within the brain region (representing signal intensity from pure blood). These synthesized CBV maps were then transformed into the template using the same transformation parameters calculated from the registration process and subsequently smoothed using a 3 mm-diameter spherical kernel. Transformed and filtered synthesized CBV maps were analyzed using SPM12 (Ashburner et al., 2014). Data were analyzed with a multiple regression model, including sex as a covariate and age as the regressor. Age-related differences were contrasted using Student's *t*-test. FreeSurfer regional segmentation was then performed on the unbiased template image, and the hippocampal formation mask is generated by binarizing and combining the labels corresponding to the hippocampus and entorhinal cortex. The age-related regression *t*-map was then projected onto the MNI-152 brain template using diffeomorphic transformation with nearest-neighbor interpolation. The result was thresholded at *p* < 0.005 and corrected for multiple comparisons at the cluster level within the hippocampal formation using a Monte-Carlo simulation implemented in AFNI-3dClustSim (Forman et al., 1995; Cox, 1996; Cox et al., 2017) (10,000 iterations) to yield a corrected *p* < 0.05. The final corrected age-related regression t-map was then overlaid onto the MNI-152 template in cross-section using 3DSlicer (Fedorov et al., 2012) and also displayed with composite-with-shading volume rendering over semi-transparent models of the hippocampal formation.

##### Pilot study part 2: Region of interest analysis on aging-related dentate gyrus region

The 177 native-space synthesized CBV scans were used to conduct the dentate gyrus (DG) region of interest (ROI) analysis. Multiple linear regression with sex as a covariate and age as the regressor was conducted over the bilateral DG, as defined by FreeSurfer parcellation. A scatter plot was drawn (**Figure 4D**) with each point representing the DG-mean synthesized CBV value after the removal of the sex effect for one participant.

##### Utility study: Synthesized CBV maps aging effects over the entire cortex

The GBCA-predicted maps were generated in the native space of each participant and were afterward used for CBV quantification together with the experimentally acquired ground truth GBCA-uptake maps using the same whole brain top 10% mean normalization. Similarly, the T1W scans were normalized to generate a comparable counterpart. We used T1W scans for comparison because they were the only input to the DeepContrast model to generate GBCA-predicted maps. The CBV (quantified from Gd-Uptake), synthesized CBV (quantified from Gd-Predicted), and normalized T1W scans were used for age-related regression in the multiple brain regions. Multiple linear regressions with sex as a covariate and age as the regressor were conducted using the mean CBV/synthesized CBV/T1W values extracted from the region across 177 participants, over selected regions (**Figure 5**) and overall 72 cortical ROIs (**Figure 6**). The ROIs were parcellated by FreeSurfer over the T1W scans in the native space in order to minimize segmentation errors.

For the ROC analysis, each ROC figure contained 1,000 individual ROC curves. The average ROC was shown as a solid black curve while the SD was shown as the shaded area. All these individual ROC curves were computed using one pair of ground truth (CBV) *t*-score maps and a prediction candidate (synthesized CBV or non-contrast T1W) *t*-score map. Both the ground truth *t*-score map and the prediction candidate *t*-score map were binarized into 2 classes at 1,000 different binarization thresholds evenly distributed between the minimum and the maximum value, yielding 1,000 versions for each. Each individual ROC curve was derived using the regular ROC computation method as described above with one of the 1,000 versions of the ground truth and all 1,000 versions of the prediction candidate. The ROC analysis was performed using Scikit-learn (Pedregosa et al., 2011).

#### Human AD

##### Pilot study part 1: Voxel-based analysis on the hippocampal circuit

Voxel-based analysis (**Figures 4E,F**) was performed by first transforming the non-contrast images using a diffeomorphic registration algorithm (Avants et al., 2009) with nearest-neighbor interpolation to an unbiased brain template created from the 200 scans (i.e., 50 AD and 50 CN participants each with 2 back-to-back repeated scans) in the pilot study. We then ran these non-contrast scans through the DeepContrast Healthy Human Model to generate synthesized CBV maps, which were subsequently smoothed using a 3 mm-diameter spherical kernel. Unlike in the aging study, the application of DeepContrast was performed after the registration process to help eliminate major anatomical variances, since the deformations present in the diseased population were not previously observed by the model trained on healthy data. GBCA-predicted scans, the direct output of the model, were used to quantify synthesized CBV maps using the same method as described in the Aging study above. These synthesized CBV maps, already co-registered upon creation, were analyzed using SPM12. Data were analyzed with a multiple regression model, including age and sex as covariates and diagnostic class (i.e., cognitive normal vs. dementia) as the regressor. AD-related differences were contrasted using Student's *t*-test. FreeSurfer regional segmentation was then performed on the unbiased template image, and the hippocampal formation mask was generated by binarizing and combining the labels corresponding to the hippocampus and the entorhinal cortex, while an extended hippocampal formation mask was additionally generated to also include the parahippocampal cortex. The AD-related regression t-map was then projected onto the MNI-152 brain template using diffeomorphic transformation with nearest-neighbor interpolation. The result was thresholded at *p* < 0.005 and corrected for multiple comparisons at the cluster level within the extended hippocampal formation using a Monte-Carlo simulation implemented in AFNI-3dClustSim (10,000 iterations) to yield a corrected *p* < 0.05. The final corrected AD-related regression t-map was then overlaid onto the MNI-152 template in cross-section using 3DSlicer and also displayed with composite-with-shading volume rendering over semi-transparent models of the hippocampal formation.

##### Pilot study part 2: Region of interest analysis on AD-related transentorhinal cortex region

The 200 template-space synthesized CBV scans were used to conduct the right transentorhinal cortex (TEC) ROI analysis. A two-sample *t*-test was conducted over the right TEC, at the boundary between the right entorhinal cortex (EC) and the right parahippocampal cortex (PHC). The region was defined as the intersection between the EC-PHC region and a sphere centered at the middle of the EC-PHC intersection and spanning a diameter of the extent of the EC-PHC boundary (11 mm). A box plot overlaid with individual data points was drawn (**Figure 4G**) to indicate the group-wise difference between the normal controls and the patients with AD.

##### Utility study: Synthesized CBV improves AD classification

For the AD classification tasks with one single input modality, the architecture “VGG-19 with batch normalization” (VGG-19BN) (Marcel et al., 2016) was used (Supplementary Figures S5a,b). When both T1W and synthesized CBV were used as input, each as one three-dimension(3D) volume, we used separate VGG encoders for each volume and later combined the extracted feature vectors before feeding them into fully-connected layers. The two encoders may have different weights (Supplementary Figure S5c). For any of these architectures, the input is the relevant 3D scans while the output is a continuous-valued number representing the predicted AD-likelihood.

To evaluate the descriptiveness of the predicted AD-likelihoods, receiver-operating characteristics (ROC) studies were conducted to analyze the concordance between the model-generated classification and the ground truth AD/CN labels. The ROC curves, one for each well-trained classifier, represent the classification performance at each potential numerical threshold to binarize the predicted AD-likelihood score. The sensitivity and specificity (the sum of whom peaks at the operating point), as well as the total area under the ROC curve, demonstrate the effectiveness of the classification method. The significance of the difference among these ROC curves is calculated using DeLong's test (DeLong et al., 1988).

Furthermore, we investigated the brain regions that had the most contributions to the AD classification task by visualizing the class activation maps (CAM) (Bolei et al., 2015). We used all 131 T1W and 131 synthesized CBV scans from patients with AD to generate an averaged CAM for each input type. We were interested in whether or not the brain regions the classifier found most relevant to the AD class were in fact physiologically meaningful.

## Results

### DeepContrast in the mouse brain

**Figure 3:**
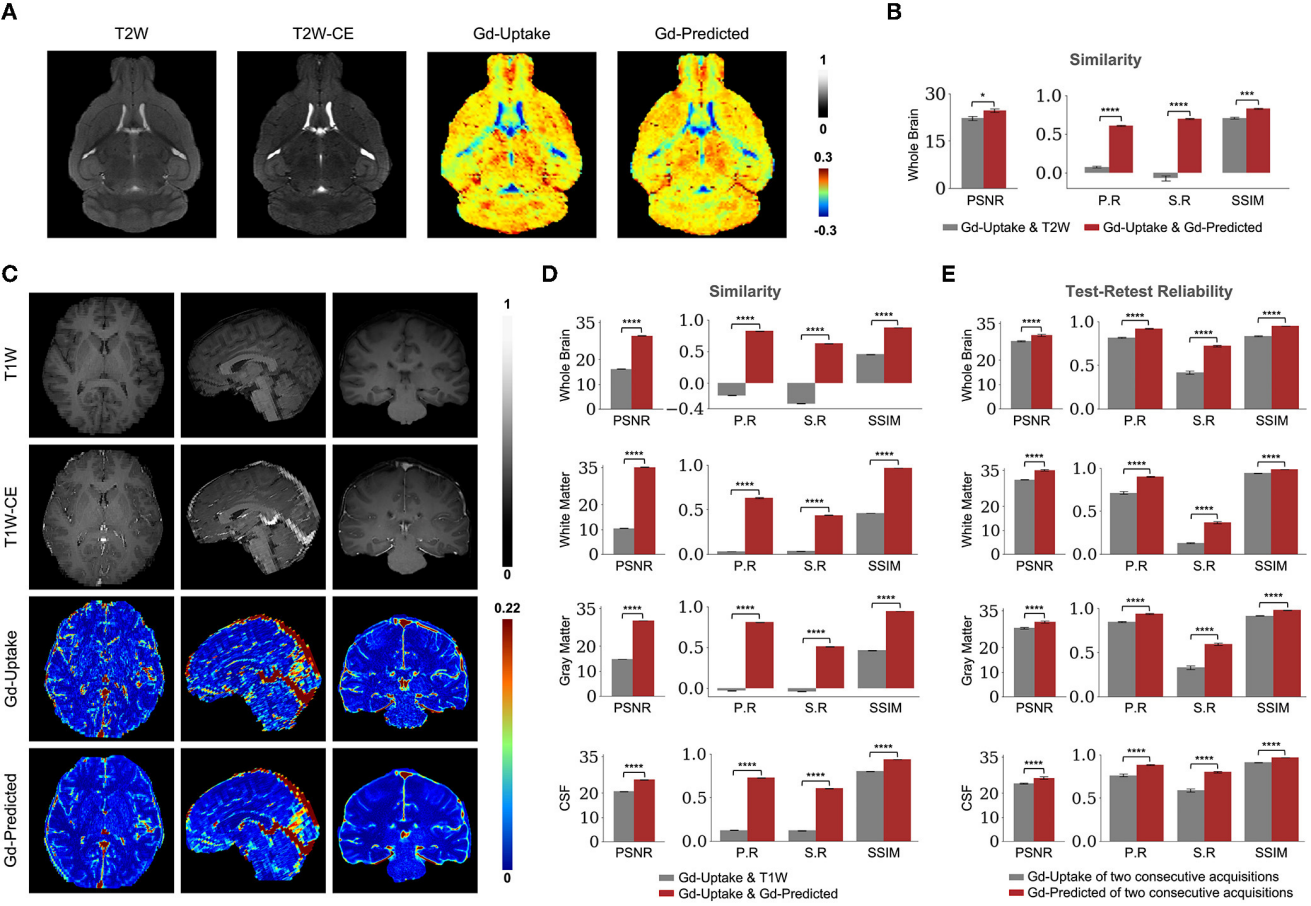
Quantitative evaluation of the DeepContrast in the healthy mouse and human brains. **(A)** DeepContrast prediction (Gd-Predicted) highly concords with the ground truth GBCA-uptake map (Gd-Uptake) in the mouse brain. The non-contrast scans and the contrast-enhanced scans are displayed for reference. Color bars indicate the colormap and dynamic range used in the cross-sectional brain images. **(B)** The similarity between the model prediction and the ground truth, evaluated on all 6 scans in the test set using quantitative metrics, where the non-contrast (T2W) scans are used as the performance baseline. **(C)** DeepContrast prediction (Gd-Predicted) highly concords with the ground truth GBCA-uptake map (Gd-Uptake) in the cognitive normal human brain. Color bars indicate the colormap and dynamic range used in the cross-sectional brain images. **(D)** The similarity between the model prediction and the ground truth, evaluated on 179 scans of cognitively normal (CN) participants using quantitative metrics, where non-contrast (T1W) scans are used as the performance baseline. **(E)** DeepContrast shows higher test-retest reliability than the experimentally acquired Gd-Uptake ground truth. For all voxel-based metrics, only the voxels within the brains or subregions are used. SSIM is calculated on the minimum bounding box of the brains or subregions. Asterisks indicate level of statistical significance (*p < 0.05, **p < 0.01, ***p < 0.001, and ****p < 0.0001). PSNR: peak signal-to-noise ratio; SSIM, structural similarity index; P. R, Pearson correlation coefficient; S.R, Spearman correlation coefficient.

We first designed, optimized, and trained the model on wildtype (WT) mice brain scans (37 for training and 6 for validation; refer to Methods section), in which we had previously generated quantitative T2-weighted GBCA-uptake brain maps. Similar to the previous studies (Kleesiek et al., 2019; Liu et al., 2019; Li et al., 2021), we compared the similarities between the GBCA-predicted maps and the GBCA-uptake ground truth maps by performing voxel-wise analyses across the whole brain on a test set with 6 scans (Figures 3A,B) using metrics that measure signal quality (peak signal-to-noise ratio) and structural similarity (structural similarity index). We further incorporated two other metrics to represent linear (Pearson correlation coefficient) and monotonic (Spearman correlation coefficient) relationships across corresponding voxels. Between the maps, the peak signal-to-noise ratio was 24.59 ± 0.60, the Pearson correlation coefficient was 0.695 ± 0.008 (*p* < 0.0001), the Spearman correlation coefficient was 0.606 ± 0.008 (*p* < 0.0001), and the structural similarity index was 0.831 ± 0.008 (Figure 3B and Table 1). This analysis shows that the DeepContrast-generated GBCA-predicted maps from WT mice showed high similarity to the GBCA-uptake ground truth maps generated from WT mice.

**Table 1:** Quantitative evaluations of DeepContrast models.

**Model**	**Evaluation**	**Region**	**Data**	**PSNR**	**P.R**	**S.R**	**SSIM**
Healthy	Similarity	Whole Brain	Gd-Uptake vs. T2W	22.16 ± 0.59	–0.072 ± 0.032	0.074 ± 0.013	0.707 ± 0.007
Mouse			Gd-Uptake vs. Gd-Predicted	24.59 ± 0.60	0.695 ± 0.008	0.606 ± 0.008	0.831 ± 0.008
Healthy	Similarity	Whole Brain	Gd-Uptake vs. T1W	15.40 ± 0.09	–0.194 ± 0.003	–0.323 ± 0.005	0.446 ± 0.002
Human			Gd-Uptake vs. Gd-Predicted	29.64 ± 0.07	0.822 ± 0.002	0.625 ± 0.003	0.879 ± 0.002
		White Matter	Gd-Uptake vs. T1W	15.40 ± 0.09	–0.194 ± 0.003	–0.323 ± 0.005	0.446 ± 0.002
			Gd-Uptake vs. Gd-Predicted	35.15 ± 0.09	0.633 ± 0.006	0.437 ± 0.002	0.969 ± 0.001
		Gray Matter	Gd-Uptake vs. T1W	14.72 ± 0.06	–0.029 ± 0.002	–0.039 ± 0.003	0.462 ± 0.001
			Gd-Uptake vs. Gd-Predicted	30.18 ± 0.07	0.807 ± 0.004	0.510 ± 0.003	0.946 ± 0.001
		CSF	Gd-Uptake vs. T1W	20.65 ± 0.07	0.124 ± 0.003	0.120 ± 0.004	0.802 ± 0.003
			Gd-Uptake vs. Gd-Predicted	25.47 ± 0.08	0.728 ± 0.004	0.604 ± 0.003	0.936 ± 0.001
	Test-Retest	Whole Brain	Gd-Uptake of two repeated acquisitions	27.70 ± 0.24	0.815 ± 0.007	0.415 ± 0.017	0.832 ± 0.007
	Reliability		Gd-Predicted of two repeated acquisitions	30.11 ± 0.44	0.919 ± 0.005	0.722 ± 0.010	0.948 ± 0.002
		White Matter	Gd-Uptake of two repeated acquisitions	31.18 ± 0.18	0.713 ± 0.015	0.129 ± 0.008	0.933 ± 0.004
			Gd-Predicted of two repeated acquisitions	35.14 ± 0.40	0.899 ± 0.006	0.368 ± 0.014	0.986 ± 0.001
		Gray Matter	Gd-Uptake of two repeated acquisitions	27.89 ± 0.32	0.844 ± 0.008	0.327 ± 0.020	0.907 ± 0.005
			Gd-Predicted of two repeated acquisitions	30.42 ± 0.44	0.937 ± 0.004	0.596 ± 0.013	0.976 ± 0.001
		CSF	Gd-Uptake of two repeated acquisitions	24.04 ± 0.27	0.762 ± 0.014	0.585 ± 0.019	0.907 ± 0.004
			Gd-Predicted of two repeated acquisitions	26.32 ± 0.48	0.882 ± 0.009	0.800 ± 0.008	0.967 ± 0.001

Evaluations varied depending on the aspects being assessed for each model. All metrics were reported in the form of mean ± standard error of the mean (SEM). PSNR, peak signal-to-noise ratio; P.R, Pearson correlation coefficient; S.R, Spearman correlation coefficient; SSIM, structural similarity index.

### DeepContrast in the human brain

We adapted the DeepContrast model to human brain MRI datasets by modifying the model network architecture, hyper-parameters, and training strategies. First, same as in our mouse study, we compared the similarities between the GBCA-predicted images or maps, and the GBCA-uptake ground truth maps by performing voxel-wise analyses across the whole brain on a test set with 179 scans (Figures 3C,D). Between the maps, the peak signal-to-noise ratio was 29.64 ± 0.07, the Pearson correlation coefficient was 0.822 ± 0.002 (*p* < 0.0001), the Spearman correlation coefficient was 0.625 ± 0.003 (*p* < 0.0001), and the structural similarity index was 0.879 ± 0.002 (Figure 3D and Table 1). Thus, in healthy human brains, we also see a high similarity between the GBCA-predicted maps and that of the GBCA-uptake ground truth maps.

In addition to the whole-brain analysis for similarity measures, we decided to extend our comparisons to two additional analyses. In the tissue of interest (TOI) analysis, we compared the similarities between the maps in white matter, gray matter, and cerebrospinal fluid (CSF). Similar to the global results, the performances by tissue types demonstrated the same trend: the GBCA-predicted maps were quantitatively similar to the GBCA-uptake ground truth maps. The results are illustrated in Figure 3D and reported in Table 1. In the region-of-interest (ROI) analysis, we compared the similarities between the maps in 126 distinct ROIs in the whole brain segmented by FreeSurfer (Fischl, 2012). Among the 126 ROIs, 121 had a significant Pearson correlation coefficients (*p* < 0.001) and 123 had significant Spearman correlation coefficients (*p* < 0.001) (Supplementary Figure S3).

Finally, we were also interested in evaluating reproducibility in a test-retest paradigm. We conducted a series of test-retest reliability analyses on the GBCA-predicted maps vs. the GBCA-uptake ground truth maps across the whole brain on a test set with 11 repeated scan pairs (Figure 3E). For the GBCA-predicted maps, the peak signal-to-noise ratio was 30.11 ± 0.44, the Pearson correlation coefficient was 0.919 ± 0.005 (*p* < 0.0001), the Spearman correlation coefficient was 0.722 ± 0.010 (*p* < 0.0001), and the structural similarity index was 0.948 ± 0.002. As a comparison, for the GBCA-uptake ground truth maps, the peak signal-to-noise ratio was 27.70 ± 0.24, the Pearson correlation coefficient was 0.815 ± 0.007 (*p* < 0.0001), the Spearman correlation coefficient was 0.415 ± 0.017 (*p* < 0.0001), and the structural similarity index was 0.832 ± 0.007. Among all the analyses, the test-retest reliabilities of the GBCA-predicted maps were consistently higher than the test-retest reliabilities of the GBCA-uptake ground truth maps (*p* < 0.0001) (Figure 3E). We also performed the TOI analysis, and the results are illustrated in Figure 3E and reported in Table 1. Among all metrics in all tissue types, the test-retest reliabilities of the GBCA-predicted maps were consistently higher than the test-retest reliabilities of the GBCA-uptake ground truth maps (*p* < 0.0001) (Figure 3E).

### DeepContrast visualizes functional lesions in aging and Alzheimer's disease brains

We generated GBCA-predicted maps from non-contrast T1-weighted MRI scans with DeepContrast, and subsequently quantified synthesized CBV maps with a sub-millimeter in-plane resolution of 0.68×0.68 mm in the coronal planes and slice thickness of 3 mm (refer to Methods). Then, we conducted voxel-based analyses (VBA) and ROI-based analyses on the synthesized CBV maps to identify sites of dysfunctions in normal aging and Alzheimer's disease (AD).

#### Normal aging

**Figure 4:**
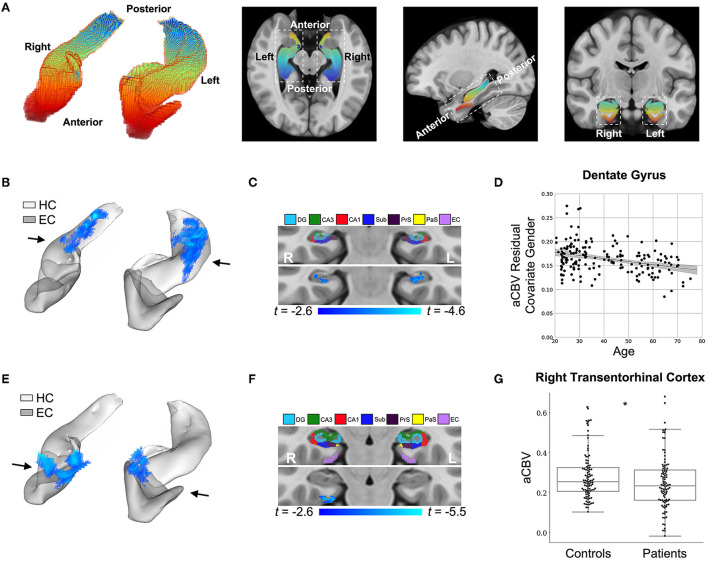
DeepContrast maps differential anatomical patterns of dysfunction in the hippocampal formation. **(A)** A three-dimensional rendering of the bilateral hippocampal formation (left panel) consisting of the hippocampus (HC) and the entorhinal cortex (EC) and axial, sagittal, and coronal slices from a group-wise T1-weighted MRI template cutting through the hippocampal formation (right three panels). The hippocampal formation is displayed along the anterior-to-posterior axis. **(B)** A voxel-based analysis of the synthesized CBV maps of 177 individuals ranging from 20 to 72 years of age reveals that the greatest age-related decline occurred in the body of the hippocampal circuit (color-coded by the degree of significance). **(C)** A coronal slice, onto which the hippocampal formation mask is applied, reveals that age-related decline primarily localizes to the dentate gyrus. The voxel-based analysis is conducted using a multiple regression model in SPM12 using sex as a covariate and age as the regressor, and the age-related differences are contrasted using Student's *t* test. Multiple comparisons are corrected for, yielding voxel-wise *p* < 0.005 and cluster-wise *p* < 0.05 (refer to methods). **(D)** A scatter plot shows the association between age and mean synthesized CBV values in the dentate gyrus after the removal of gender effects (β_*age*_ = −6.36e-4, *t*_*age*_ = −4.64, *p*_*age*_ = 6.85e-6). The shaded area surrounding the regression line indicates the 95% CI. **(E)** A voxel-based analysis of the synthesized CBV maps of 50 Alzheimer's disease (AD) patients compared with 50 age-matched normal controls, each with two back-to-back scans, reveals AD-related reduction in the entorhinal cortex (color-coded by the degree of significance). **(F)** A coronal slice, onto which the hippocampal formation mask is applied, reveals that AD-related decline localizes primarily to the transentorhinal cortex. The voxel-based analysis is conducted using a multiple regression model in SPM12 using age, sex, and participant identity as covariates and diagnostic class (i.e., cognitive normal vs. dementia) as the regressor and the AD-related difference are contrasted using Student's *t*-test. Multiple comparisons are corrected for, yielding voxel-wise *p* < 0.005 and cluster-wise *p* < 0.05 (refer to methods). **(G)** A box plot showing individual-participant mean synthesized CBV values in the right transentorhinal cortex indicates a significant difference between patients with Alzheimer's disease and healthy controls (two sample *t*-test one-tailed *p* = 0.031). Center line: median; box limits: upper and lower quartiles; whiskers: 1.5× interquartile range; points: outliers. HC: hippocampus; EC: entorhinal cortex; DG: dentate gyrus; CA3: *cornu Ammonis* 3; CA1: *cornu Ammonis* 1; Sub: subiculum; Prs: presubiculum; PaS: parasubiculum.

The first study we conducted aimed to validate whether DeepContrast can capture the subtle aging effects on basal metabolism. First, we focused on the hippocampal circuit (Figure 4A). As shown in Figures 4B,C, the age-related decline in our DeepContrast-generated synthesized CBV maps localized primarily to the dentate gyrus (DG). This result replicates prior studies (Small et al., 2002, 2004; Chawla and Barnes, 2007; Moreno et al., 2007; Brickman et al., 2014; Feng et al., 2020a), where the age-related decline in brain metabolism in the hippocampal formation has been shown to occur primarily in the DG. In the complementary ROI analysis of the DG, the synthesized CBV values showed a linear decline with age (β_*age*_ = −6.36e-4, *t*_*age*_ = −4.64, *p*_*age*_ = 6.85e-6) (Figure 4D).

**Figure 5:**
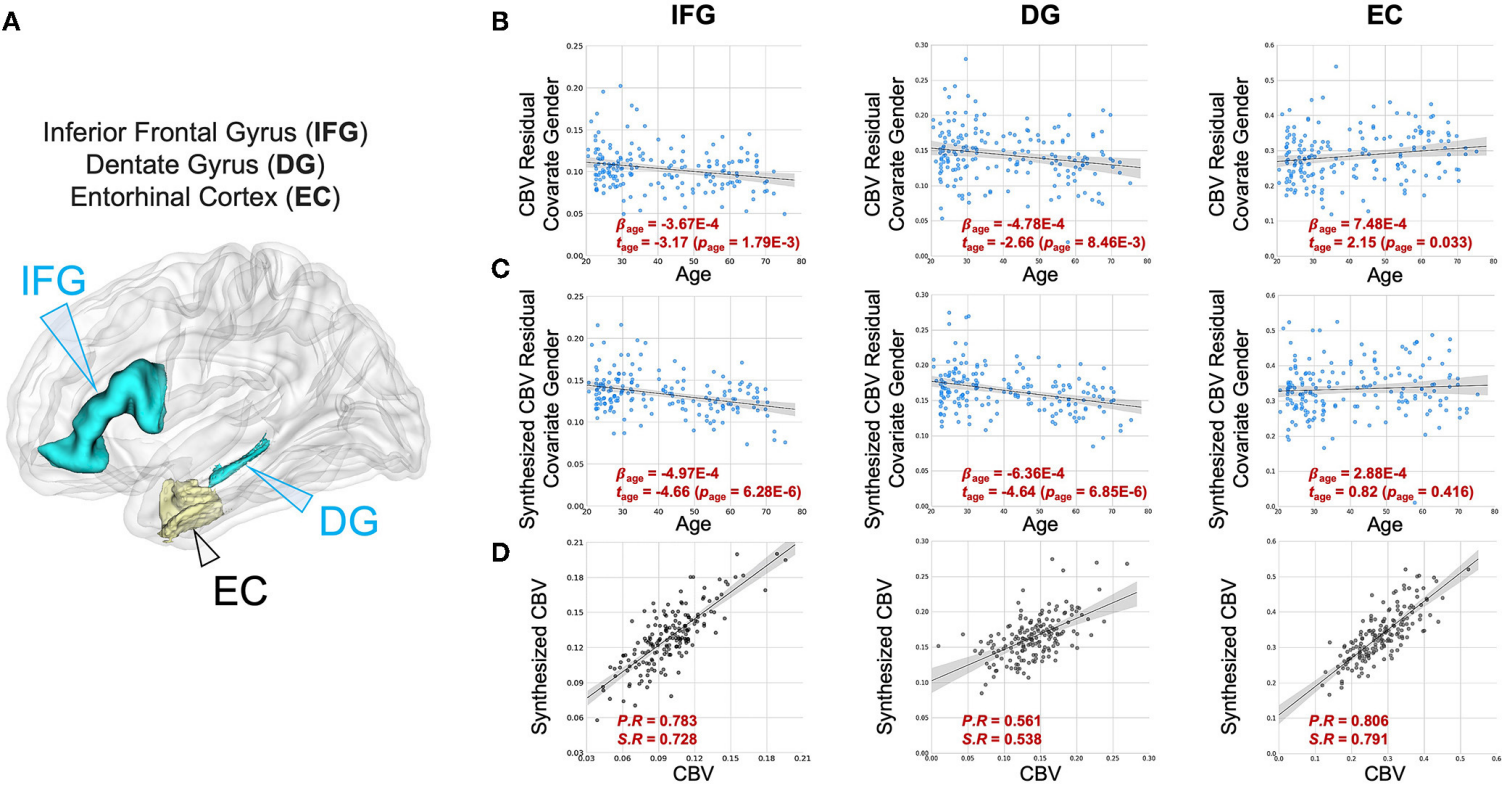
DeepContrast maps age-related changes in brain regions vulnerable and resistant to aging. **(A)** A three-dimensional rendering of the inferior frontal gyrus (IFG), dentate gyrus (DG) and entorhinal cortex (EC) overlaid on a group-wise T1-weighted MRI template. **(B)** The age-related regressions of CBV maps over these regions demonstrate the aging-vulnerability of IFG and DG and the aging-resistance of EC. **(C)** The age-related regressions of synthesized CBV maps over the same regions demonstrated the same vulnerability or resistance to aging. **(D)** The scatter plots of the ROI-mean CBV vs. synthesized CBV values of the 177 participants further show the consistency of the two measures.

We also analyzed two other brain regions, namely the inferior frontal gyrus (IFG), found to be more vulnerable to aging (Shen et al., 2012; Hoffman and Morcom, 2018; Feng et al., 2020a,b), and the entorhinal cortex (EC), found to be less vulnerable to aging (Gómez-Isla et al., 1996; Small et al., 2004, 2011; Feng et al., 2020a). The synthesized CBV maps demonstrated the same age-related trends as the ground truth CBV over these regions (Figure 5).

**Figure 6:**
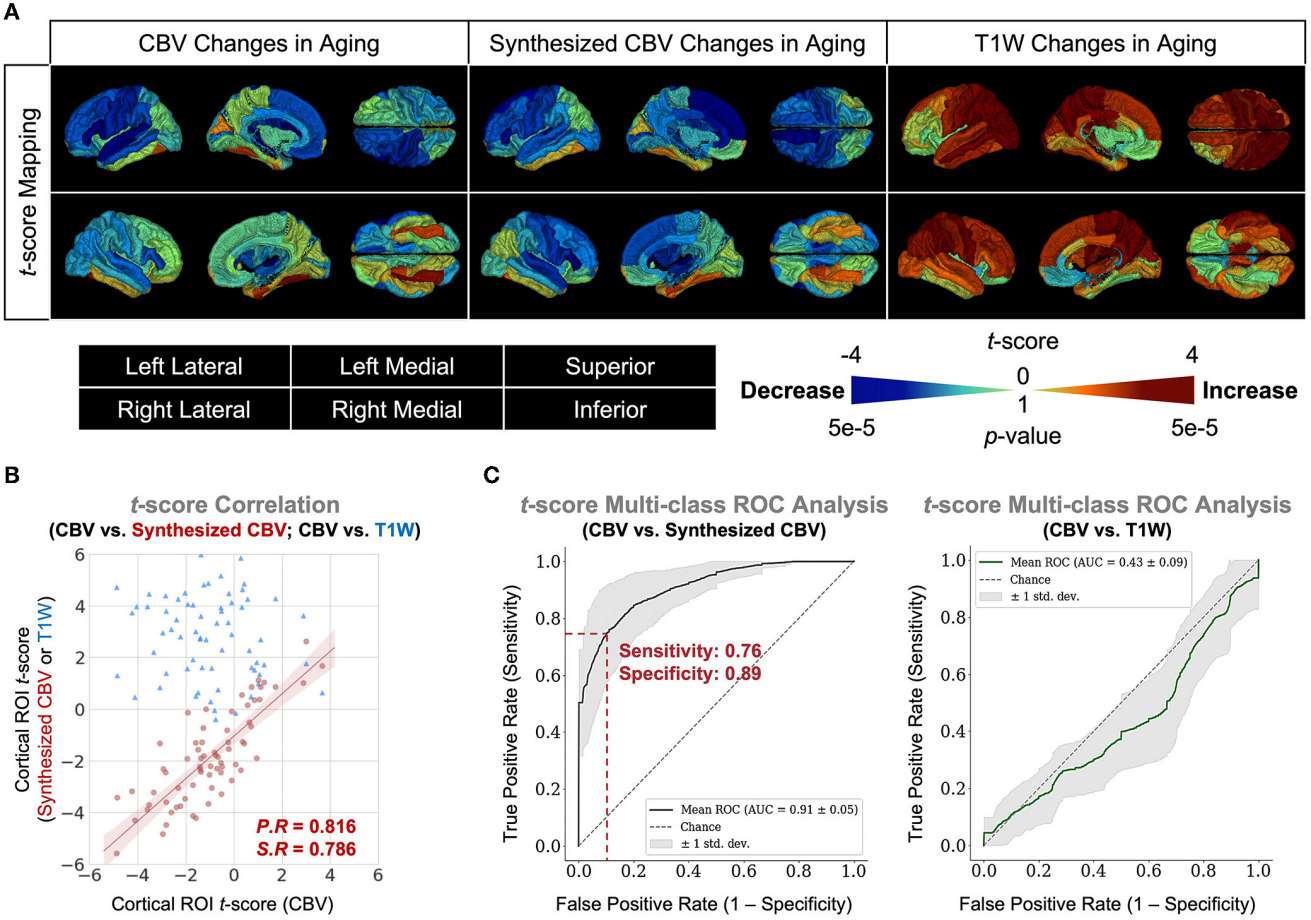
DeepContrast maps age-related changes over the entire cortex. **(A)** Three-dimensional volume rendering of the age-related *t*-score maps over the 72 FreeSurfer cortical region-of-interests (ROIs) reveals that the age-related changes in the synthesized CBV maps is similar to those in the ground truth CBV maps, despite that the non-contrast T1W scans which serves as the input to the DeepContrast model does not share either the same or the opposite trends. **(B)** A scatter plot of the age-related *t*-score over the 72 ROIs demonstrates that the age-related changes in synthesized CBV are consistent to those in CBV (P.R = 0.816, S.R = 0.786) while the T1W counterparts do not (P.R = –0.131, S.R = –0.122). **(C)** An analysis of the concordance to CBV *t*-scores by treating it as a 1,000-class classification problem reveals that age-related changes in synthesized CBV have significant predictive power on those in CBV (sensitivity = 0.76, specificity = 0.89, AUC = 0.91) while the T1W counterparts do not (sensitivity = 1.00, specificity = 0.04, AUC = 0.43).

Finally, we extended the analysis to the entire cortex and found that the synthesized CBV maps reflected similar age-related changes as the ground truth CBV overall cortical ROIs (Figure 6). The multi-class Receiver Operative Characteristics (ROC) curve, which represented the level of concordance between synthesized CBV and ground truth CBV, reached a sensitivity of 0.76 and a specificity of 0.89 at the operating point, and the area-under-the-curve (AUC) was 0.91 (Figure 6).

#### Alzheimer's disease

The second study we conducted aimed to validate whether DeepContrast could capture the regional vulnerability in patients with Alzheimer's disease dementia, where we utilized publicly available data from the Alzheimer's Disease Neuroimaging Initiative (ADNI) (Mueller et al., 2005). Similar to the above approach, we performed a VBA analysis over the hippocampal circuit (Figure 4A). Replicating previous findings (Braak and Braak, 1996; Brickman et al., 2011, 2014; Small et al., 2011; Pavlopoulos et al., 2013; Schobel et al., 2013; Khan et al., 2014; Sperling et al., 2014; Coughlan et al., 2018; Provenzano et al., 2020; Simoes et al., 2021), the Alzheimer's disease-related decline in the synthesized CBV maps primarily localized to a region termed the transentorhinal cortex (TEC) (Figures 4E,F). In the complementary ROI analysis of the right TEC, the synthesized CBV values were significantly lower (*p* = 0.031) in patients with AD compared to the healthy controls (Figure 4G).

**Figure 7:**
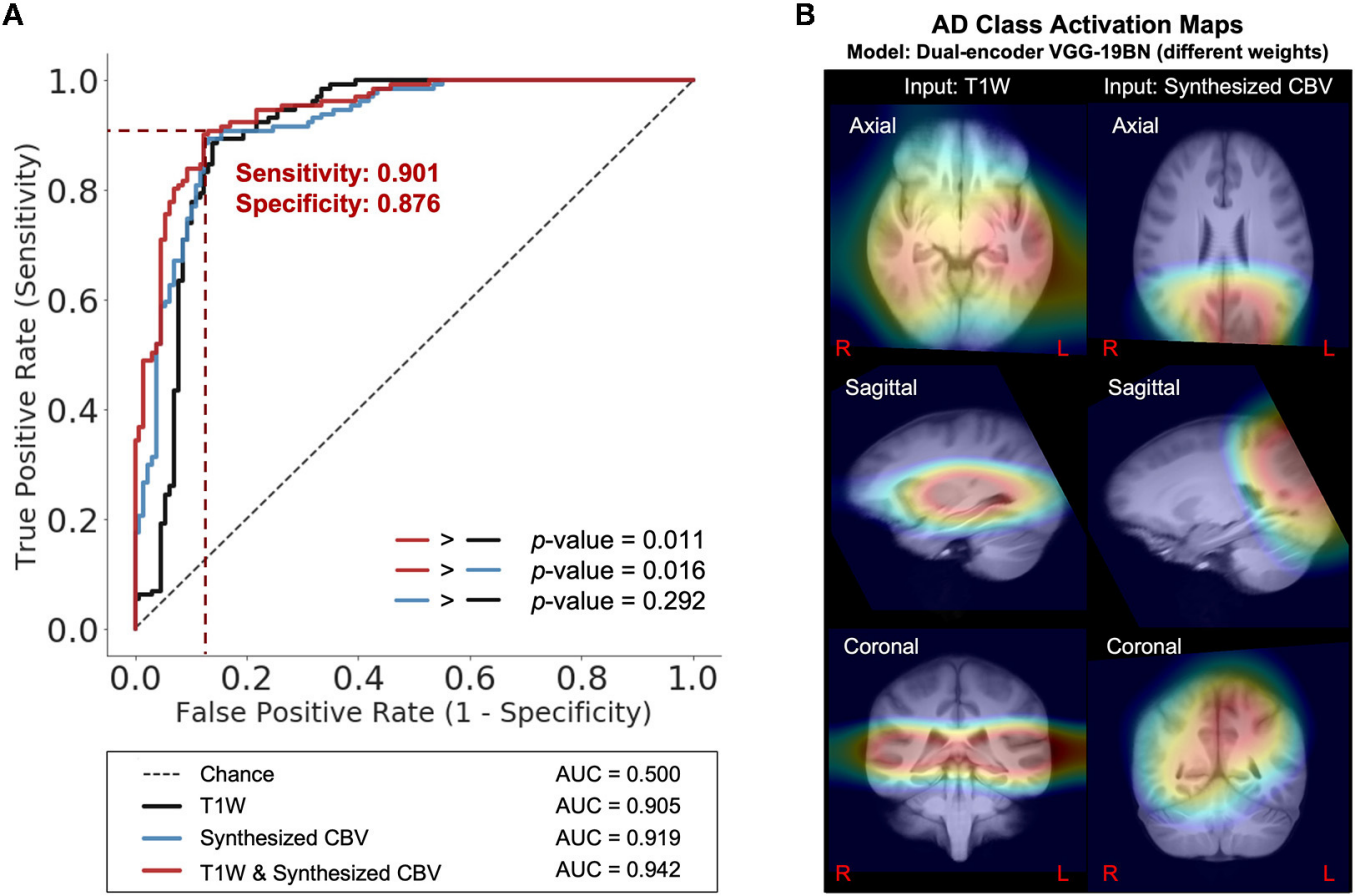
DeepContrast-generated synthesized CBV improves AD classification and provides spatially-meaningful information. **(A)** Receiver Operating Characteristics curves for the classification models using T1W structural data only (black), using synthesized CBV functional data only (blue), or using both in combination (red). *p*-values were calculated using DeLong's test. **(B)** The class-average Class Activation Maps of the best-performing model, calculated from 131 AD scans in the standalone test set.

Next, we trained VGG-like models (Simon et al., 2016) to perform participant-level AD classification on a class-balanced and age-matched dataset with more than 2,500 scans. We tested the models on the same stand-alone set of 131 AD scans and 129 CN scans. Compared to using the T1W MRI data alone, when we included the synthesized CBV maps generated using DeepContrast as the input to the model, the classification accuracy increased significantly (Figure 7A). However, the specific approach to combining the two modalities affected the performances (Table 2). The best fit that we found was to assign a designated encoder for each modality without weight sharing between them.

**Table 2:** Performances of the five variants of the AD-classification network.

**Input**	**Model**	**Sensitivity**	**Specificity**	**ROC AUC**	**Accuracy @ Operating**	**Max accuracy**
T1W	Regular	0.885	0.860	0.905	0.869	0.873
SynCBV	Regular	0.885	**0.876**	0.919	0.877	0.881
T1W+SynCBV	Dual-channel	0.885	0.853	0.936	0.865	0.869
T1W+SynCBV	Dual-encoder w/ identical weights	0.802	0.806	0.875	0.800	0.804
T1W+SynCBV	Dual-encoder w/ different weights	**0.901**	**0.876**	**0.942**	**0.885**	**0.888**

Sensitivity and specificity are calculated at the operating point. Accuracy at the operating point and the maximum accuracy achievable by changing the binarization threshold are respectively calculated for each candidate. ROC AUC, area under the receiver-operating characteristics curve; SynCBV, synthesized CBV. Best result(s) in each metric are highlighted in bold.

Finally, we used class activation maps to identify specific brain regions that influenced the best performing classifier when it determined that a person has AD (Figure 7B). The most highly contributing structural information comes from the temporal lobe, while the most highly informative artificial functional information was observed to come from the parieto-occipital lobe.

## Discussion

Gadolinium-based contrast agents's utility for MRI can be organized around two primary pathophysiologies. The first is a breakdown of the blood-brain barrier that often accompanies many structural lesions, and, in that case, GBCA extravasates into the parenchyma and enhances lesion detection (Garcia et al., 2020). The second is alterations in neuronal metabolism, typical of most functional disorders, in which case intravascular GBCA is used to quantify regional CBV, a cerebrovascular variable tightly coupled to energy metabolism (Belliveau, 1991; Gonz et al., 1995; Østergaard et al., 1998; Sugahara et al., 1998; Aronen et al., 2000). As proof-of-principle, we optimized models for our investigation of the second scenario. We have demonstrated that DeepContrast can extract GBCA-like contrast information from non-contrast T1W structural MRI scans to quantify regional CBV. As GBCA's utility can be largely divided into two pathophysiologies, we anticipate that future large-scale studies across a range of diseases might lead to two generalizable models–one for structural disorders that are more likely to impact the integrity of the blood-brain barrier, and another for functional lesions that alters brain metabolism, although GBCA contrast is much subtler for functional compared to structural lesions.

DeepContrast's utility can be organized according to its broad applications. The first is for research. There is an increasing number of brain MRI databases, such as ADNI, whose primary purpose is brain imaging and disease research. Standard T1-weighted MRI scans are among the most common protocols across all of these datasets, typically acquired for mapping regional structural differences, such as regional volume or cortical thickness. DeepContrast can be retroactively applied to these data and can be used to generate synthetic functional maps, significantly expanding pathophysiological insight that can be derived across a range of disorders. For example, by using the DeepContrast model, we have demonstrated that a large-scale synthetic functional dataset could be generated and further used to provide superior AD classification. For single input modalities, the AD classifier trained on the synthesized CBV functional images provided improved AD identification when compared with the AD classifier trained on T1W scans. The improvement was further amplified when both modalities were provided, which allowed the model to take advantage of both structural and functional information. Among all the candidate models, the model with two separate encoders of different weights outperforms the others. Training each encoder on structural and synthesize functional CBV MRI scans independently allowed the most efficient feature extraction and yielded the best classification performances. The class activation map for the best-performing model revealed an interesting pattern of collaboration between the two encoders, each corresponding to a single input modality. The medial temporal lobe provides the most crucial structural information as reflected by the structural-encoder. This result is consistent with previous studies indicating that medial temporal atrophy is an indicative sign of AD and qualitative assessments of the region could be used to predict patients at risk of AD (Bradley et al., 1984; Korf et al., 2004). On the other hand, activation of the parietal and occipital lobes was representative of regions experiencing the most functional changes in the AD brain in accordance with the functional-encoder, which is consistent with the findings such as decreased resting state neural activity (Yong et al., 2007; Li et al., 2016) and glucose utilization (Reiman et al., 1996) in the parieto-occipital cortex.

DeepContrast's second application is for patient care. For patient populations with functional lesions, those with neuropsychiatric and neurodegenerative disorders, a T1-weighted scan may be ordered as part of standard clinical practice, to exclude structural abnormalities. For these patients, deriving CBV maps*via* DeepContrast potentially obviates the need for ordering other more invasive, burdensome, and expensive neuroimaging studies for mapping metabolic dysfunction.

This study has several limitations. First, our study focused on the identification of functional abnormalities with the synthesized CBV derived from non-contrast T1W structural MRI without the need for GBCAs. Detecting structural brain lesions such as those found in cancer, infections, or bleeding are still the major utility of GBCAs. Our DeepContrast framework is sufficiently general that it can be easily extended to the detection of structural brain lesions with T1W MRIs, but future study should be done to evaluate its performance in these conditions. Second, we acknowledge the retrospective nature of the study and the absence of inclusion of MRI data from multiple sites and acquisition protocols. The distinct quantitative GBCA dataset used to train our DeepContrast model was collected over a timeframe of around 20 years at Columbia University using the equivalent protocol on multiple scanners. The pre- and post-GBCA images used to derive the CBV maps shared identical imaging settings, which help reduce the inter and intrasubject variability between the non-contrast T1W pre-GBCA image and the quantitative GBCA enhancement and benefit the training of our model. Future study could address how the use of heterogeneous data from various cohorts, sites, scanners, and acquisition protocols might improve the model performance to produce more stable and generalizable results. This study also shares the limitations of other studies of GBCA contrast synthesizing with deep learning. Deep convolutional neural networks have performed remarkably well on these tasks; however, these networks are heavily reliant on big data to avoid overfitting. Unfortunately, medical image analysis applications normally do not have access to big data. Data augmentation, a data-space solution to the problem, of limited data can be applied in future work to enhance the sample size, enrich the data variance and improve the data quality of the training dataset such that better models can be built from them. Finally, in light of the overall promising performance on the task, it is important to consider our work as a pioneer proof-of-concept study, and future work should be done to further improve its performance to reach the level for clinical purposes. For instance, the proposed CNN model is trained from scratch, but applying and fine-tuning a pre-trained model on our data through transfer learning could further improve model performance. Future study could also improve our model through multi-task learning. While we did achieve state-of-the-art performance by being laser-focused on our single task, synthesizing CBV from non-contrast T1W structural MRI, we ignored information that might help us achieve even better metrics. Specifically, this information could come from training MRI signals for related tasks, such as image reconstruction, brain tissue segmentation, or predicting demographic information. By sharing representations between related tasks in a multi-task learning framework, it could enable our model to generalize better on the original task.

In conclusion, by using quantitative GBCA datasets from both mice and humans, we demonstrated that a deep learning model can, in principle, generate GBCA-equivalent information from a single structural MRI scan for the estimation of regional CBV, and we successfully applied our DeepContrast model to both an in-house aging dataset and a publicly available ADNI dataset from Alzheimer's patients and age-matched controls.

## Data availability statement

The trained Healthy Human Model, alongside the test-retest reliability dataset (*n* = 11, each with two test-retest acquisitions) with both non-contrast scans and ground truth GBCA-uptake maps, is available on GitHub (https://github.com/SAIL-GuoLab/DeepContrast_Demo). The scripts that predict GBCA-uptake maps from non-contrast scans, as well as the script performing quantitative evaluations, are included. All code and data (except for those from public datasets) are proprietary and managed by the Columbia Technology Ventures Office of Intellectual Property. The custom training code and large-scale datasets are not publicly available. Further inquiries can be directed to the corresponding author.

## Ethics statement

The animal study was reviewed and approved by the Institutional Animal Care and Use Committee (IACUC), NIH.

## Author contributions

JG conceived, designed, and supervised all studies constituting this article and verified all statistical results. CL and NZ participated in the design and optimization of all DeepContrast models, and particularly optimized and trained the healthy human model including data preprocessing, performed statistical analyses of the Healthy Human Model, conducted the Human Aging study, and conducted the Human AD study. HS and XL led the optimization and training of the Healthy Mouse Model including data preprocessing. SS kindly provided all human MRI data in the Columbia University cohort, which was collected, organized, and maintained by FP and XF kindly provided the organized and preprocessed ADNI data, and the brain parcellations of the 177 healthy participants used in the Human Aging study. JG and SG-S participated in the acquisition of the mouse MRI data. HS performed statistical analyses of the healthy mouse model. CL, NZ, SS, FP, TN, and JG wrote the manuscript. NZ, CL, and JZ created and updated all display items (figures and tables) and supplementary information (figures and tables). All authors reviewed, commented, and edited the manuscript. Data used in the Human AD study was obtained from the ADNI database (adni.loni.usc.edu). As such, the investigators within the ADNI contributed to the design and implementation of ADNI and/or provided data but did not participate in the analysis or writing of this manuscript.

## Funding

This study was funded by the Seed Grant Program and Technical Development Grant Program at the Columbia MR Research Center. This study was also funded by grants from Alzheimer's Disease Research Center (P30AG066462), Matheson Foundation (UR010590), and the cancer center support grant (P30CA013696 NIH/NIC). This study was performed at the Zuckerman Mind Brain Behavior Institute MRI Platform, a shared resource. Data collection and sharing for this project was partially funded by the Alzheimer's Disease Neuroimaging Initiative (ADNI) (National Institutes of Health Grant U01 AG024904) and DOD ADNI (Department of Defense award number W81XWH-12-2-0012). ADNI is funded by the National Institute on Aging, the National Institute of Biomedical Imaging and Bioengineering, and through generous contributions from the following: AbbVie, Alzheimer's Association; Alzheimer's Drug Discovery Foundation; Araclon Biotech; BioClinica, Inc.; Biogen; Bristol-Myers Squibb Company; CereSpir, Inc.; Cogstate; Eisai Inc.; Elan Pharmaceuticals, Inc.; Eli Lilly and Company; EuroImmun; F. Hoffmann-La Roche Ltd and its affiliated company Genentech, Inc.; Fujirebio; GE Healthcare; IXICO Ltd.; Janssen Alzheimer Immunotherapy Research & Development, LLC.; Johnson & Johnson Pharmaceutical Research & Development LLC.; Lumosity; Lundbeck; Merck & Co., Inc.; Meso Scale Diagnostics, LLC.; NeuroRx Research; Neurotrack Technologies; Novartis Pharmaceuticals Corporation; Pfizer Inc.; Piramal Imaging; Servier; Takeda Pharmaceutical Company; and Transition Therapeutics. The Canadian Institutes of Health Research is providing funds to support ADNI clinical sites in Canada. Private sector contributions are facilitated by the Foundation for the National Institutes of Health (www.fnih.org). The grantee organization is the Northern California Institute for Research and Education, and the study is coordinated by the Alzheimer's Therapeutic Research Institute at the University of Southern California. ADNI data are disseminated by the Laboratory for Neuro Imaging at the University of Southern California.

## Conflict of interest

Author FP is a consultant for and equity holder of Imij Technologies. Author SS serves on the scientific advisory board of Meira GTX, recently came off the scientific advisory board of Denali Theraputics, and is an equity holder in Imij Technologies. Authors XF, FP, SS, and JG have either granted patents or applications in neuroimaging for which no royalties are received. The remaining authors declare that the research was conducted in the absence of any commercial or financial relationships that could be construed as a potential conflict of interest.

## Publisher's note

All claims expressed in this article are solely those of the authors and do not necessarily represent those of their affiliated organizations, or those of the publisher, the editors and the reviewers. Any product that may be evaluated in this article, or claim that may be made by its manufacturer, is not guaranteed or endorsed by the publisher.

## Supplementary material

Supplementary Figure S1 Details of the Healthy Mouse Model (Network 1 in Figure 2). This model is implemented with a 2D five-layered Residual Attention U-Net architecture. The encoding path (blue blocks in the left half of the architecture) condenses the image dimensions and enriches the feature dimension, shrinking the image size from 208×208 pixels to 13×13 pixels while extracting 1,024 channels of features. The decoding path (red blocks in the right half of the architecture) expands these high-level features and returns back a single slice of the predicted image with 208×208 pixels.

Supplementary Figure S2 Details of the Healthy Human Model (Network 2 in Figure 2). This model is implemented with a 2D six-layered Residual Attention U-Net architecture. The encoding path (blue blocks in the left half of the architecture) condenses the image dimensions and enriches the feature dimension, shrinking the image size from 352×352 pixels to 11×11 pixels while extracting 2,048 channels of features. The decoding path (red blocks in the right half of the architecture) expands these high-level features and returns back a single slice of the predicted image with 352×352 pixels.

Supplementary Figure S3 Correlation between the GBCA-uptake map and GBCA-predicted map over the entire brain. The Pearson (top) and Spearman (bottom) correlation coefficients are computed for each of the 126 anatomical ROIs defined by FreeSurfer between the array of ROI-mean Gd-Uptake values and the array of ROI-mean Gd-Predicted values extracted from the 177 participants with successful FreeSurfer parcellation. The analysis demonstrates significant correlation between the DeepContrast-predicted contrast and the corresponding ground truth across a wide range of brain regions (121 ROIs with *p* < 0.001 for Pearson correlation coefficient and 123 ROIs with p < 0.001 for Spearman correlation coefficient).

Supplementary Figure S4 Details of MRI processing pipeline and partition of the Alzheimer's Disease (AD) classification dataset. **(a)** Data processing pipeline to generate the input of different AD classification deep learning models. The preprocessing of structural T1W MR data is necessary to remove unwanted artifacts and transform the data into a standard version before feeding them into the models. For each MRI structural, we process the T1W 3D volume through a standardized pipeline: (1) whole brain affine registration to MNI152 template space and other processing detailed in a prior study (Feng et al., 2018); (2) whole brain rigid registration to MNI152 template space; (3) histogram matching to the DeepContrast CU T1W MRI; (4) whole brain diffeomorphic registration to standard CU template; (5) generating the synthesized CBV maps; (6) up-sampling the synthesized CBV maps to 1-mm isotropic resolution. **(b)** Left: Age distributions of the participants in the entire dataset (top) and the subset after propensity-score matching of age (bottom). Right: Age distributions of the participants assigned to the train, validation, and test dataset. VS, voxel size; AD, Alzheimer's diseased; CN, cognitive normal.

Supplementary Figure S5 Details of the three candidates of AD-classification networks (Networks used in Figure 7). **(a)** Proposed Model 1. Modified 3D VGG-19 network with batch normalization uses T1W MRI scans as the model input. This model aims to perform the AD classification based on structure T1W MRI scans. **(b)** Proposed Model 2. Modified 3D VGG-19 network with batch normalization uses synthesized CBV maps as the model input. This model aims to realize the AD classification based on functional MRI maps. **(c)** Proposed model 3. Modified VGG-19 network with double encoding paths uses T1W MRI scans and the synthesized CBV maps as the model input. Separate encoders in the model have the same structure but different weights. This could help improve the structure and functional information fusion and classification performance. The red arrow represents the generation of synthesized CBV from T1W MRI scans using DeepContrast.
