# Supplementary figures and images for: Deep learning of MRI contrast enhancement for mapping cerebral blood volume from single-modal non-contrast scans of aging and Alzheimer's disease brains
Source: Front Aging Neurosci. 2022 Aug 11;14:923673. doi: 10.3389/fnagi.2022.923673 (PMC9407020; doi:10.3389/fnagi.2022.923673)

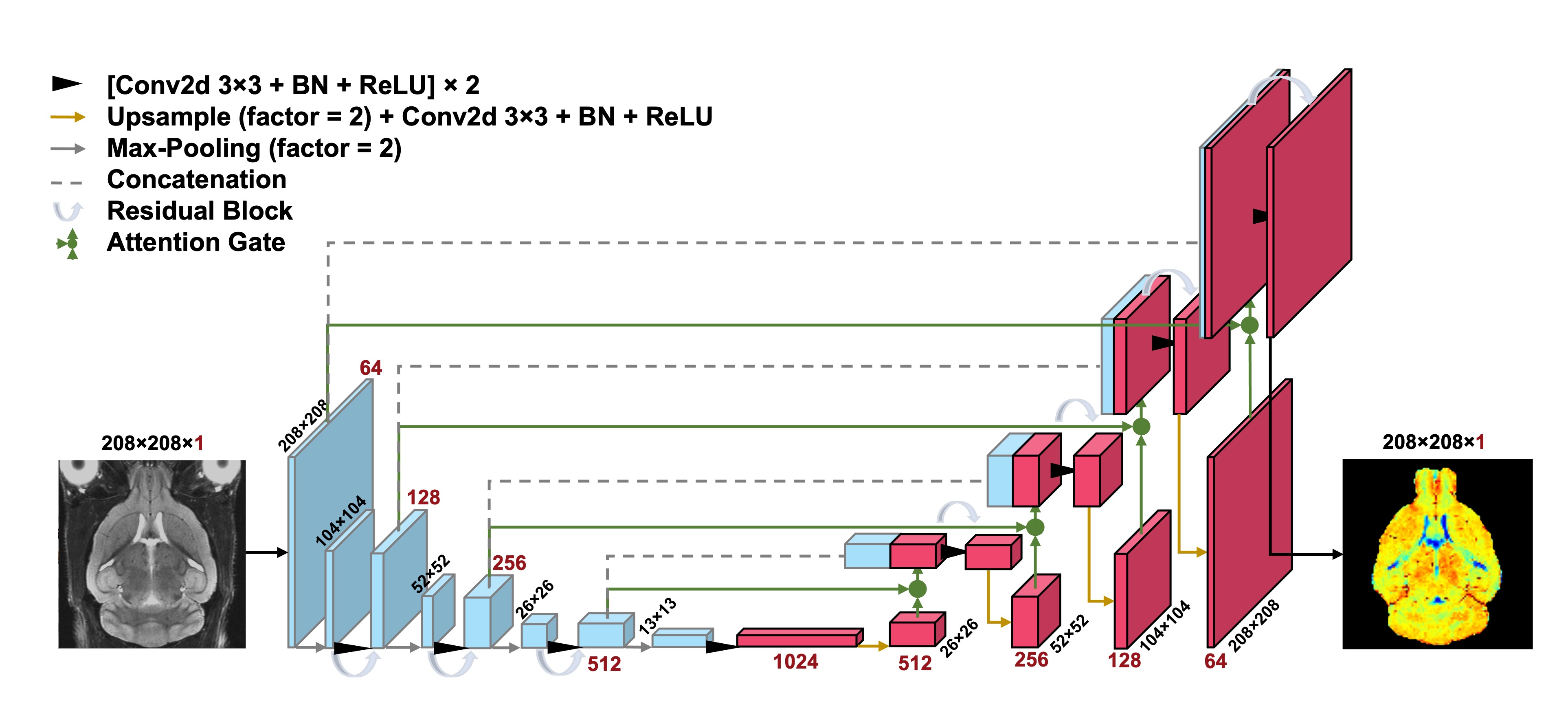

Supplement: Supplementary Figure S1 — Details of the Healthy Mouse Model (Network 1 in Figure 2). This model is implemented with a 2D five-layered Residual Attention U-Net architecture. The encoding path (blue blocks in the left half of the architecture) condenses the image dimensions and enriches the feature dimension, shrinking the image size from 208×208 pixels to 13×13 pixels while extracting 1,024 channels of features. The decoding path (red blocks in the right half of the architecture) expands these high-level features and returns back a single slice of the predicted image with 208×208 pixels. [file Image_1.JPEG]

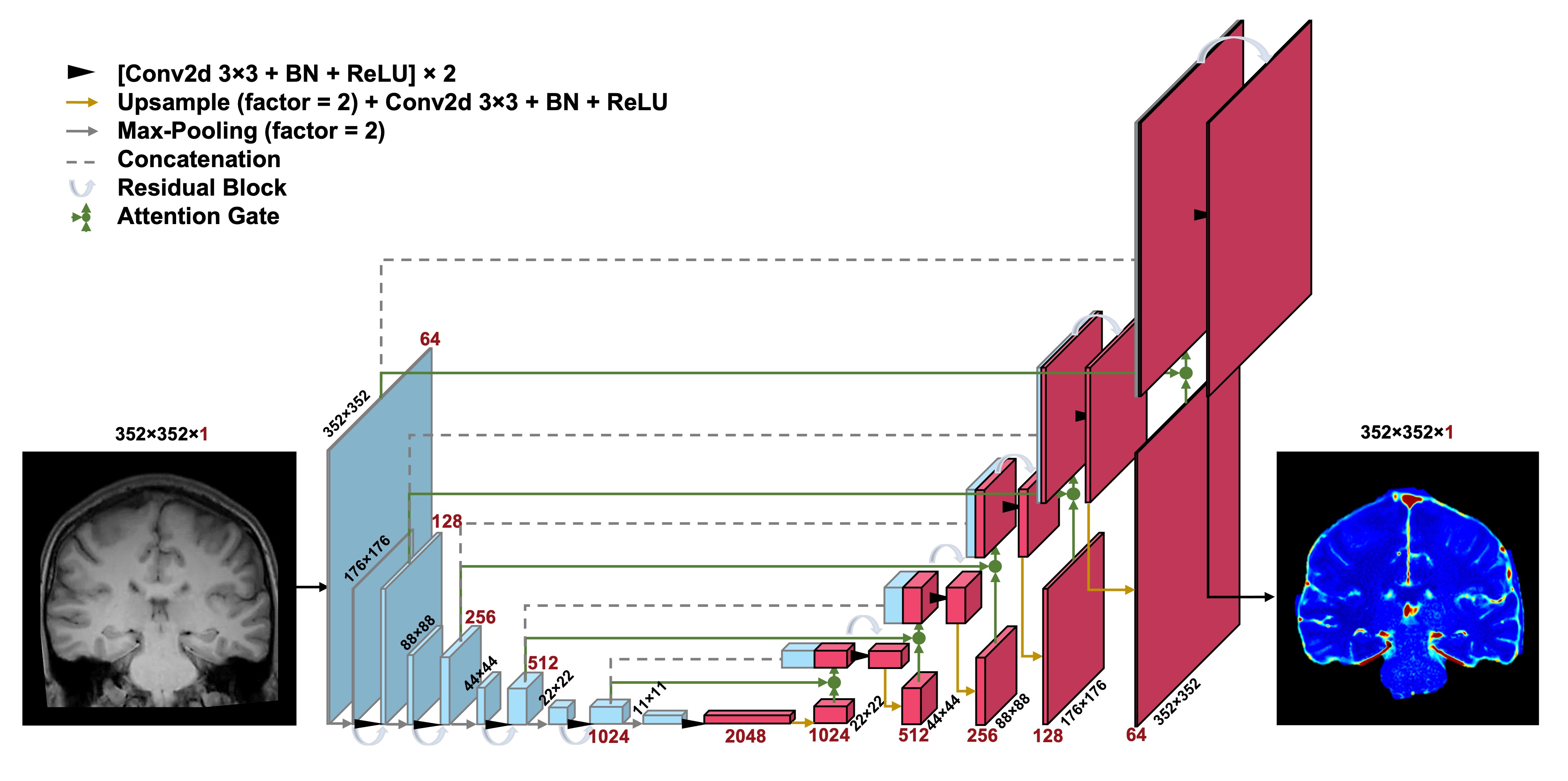

Supplement: Supplementary Figure S2 — Details of the Healthy Human Model (Network 2 in Figure 2). This model is implemented with a 2D six-layered Residual Attention U-Net architecture. The encoding path (blue blocks in the left half of the architecture) condenses the image dimensions and enriches the feature dimension, shrinking the image size from 352×352 pixels to 11×11 pixels while extracting 2,048 channels of features. The decoding path (red blocks in the right half of the architecture) expands these high-level features and returns back a single slice of the predicted image with 352×352 pixels. [file Image_2.JPEG]

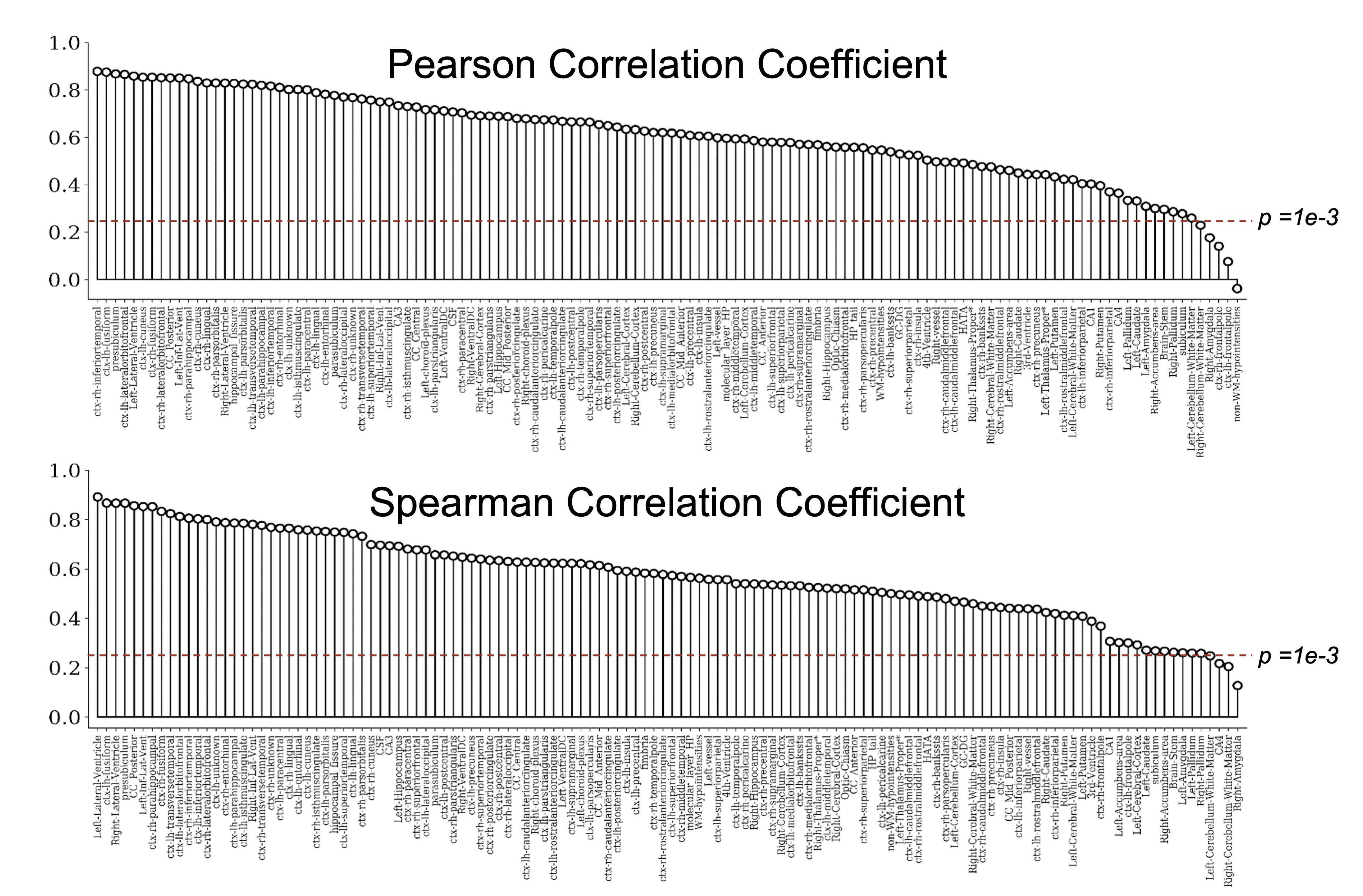

Supplement: Supplementary Figure S3 — Correlation between the GBCA-uptake map and GBCA-predicted map over the entire brain. The Pearson (top) and Spearman (bottom) correlation coefficients are computed for each of the 126 anatomical ROIs defined by FreeSurfer between the array of ROI-mean Gd-Uptake values and the array of ROI-mean Gd-Predicted values extracted from the 177 participants with successful FreeSurfer parcellation. The analysis demonstrates significant correlation between the DeepContrast-predicted contrast and the corresponding ground truth across a wide range of brain regions (121 ROIs with p < 0.001 for Pearson correlation coefficient and 123 ROIs with p < 0.001 for Spearman correlation coefficient). [file Image_3.JPEG]

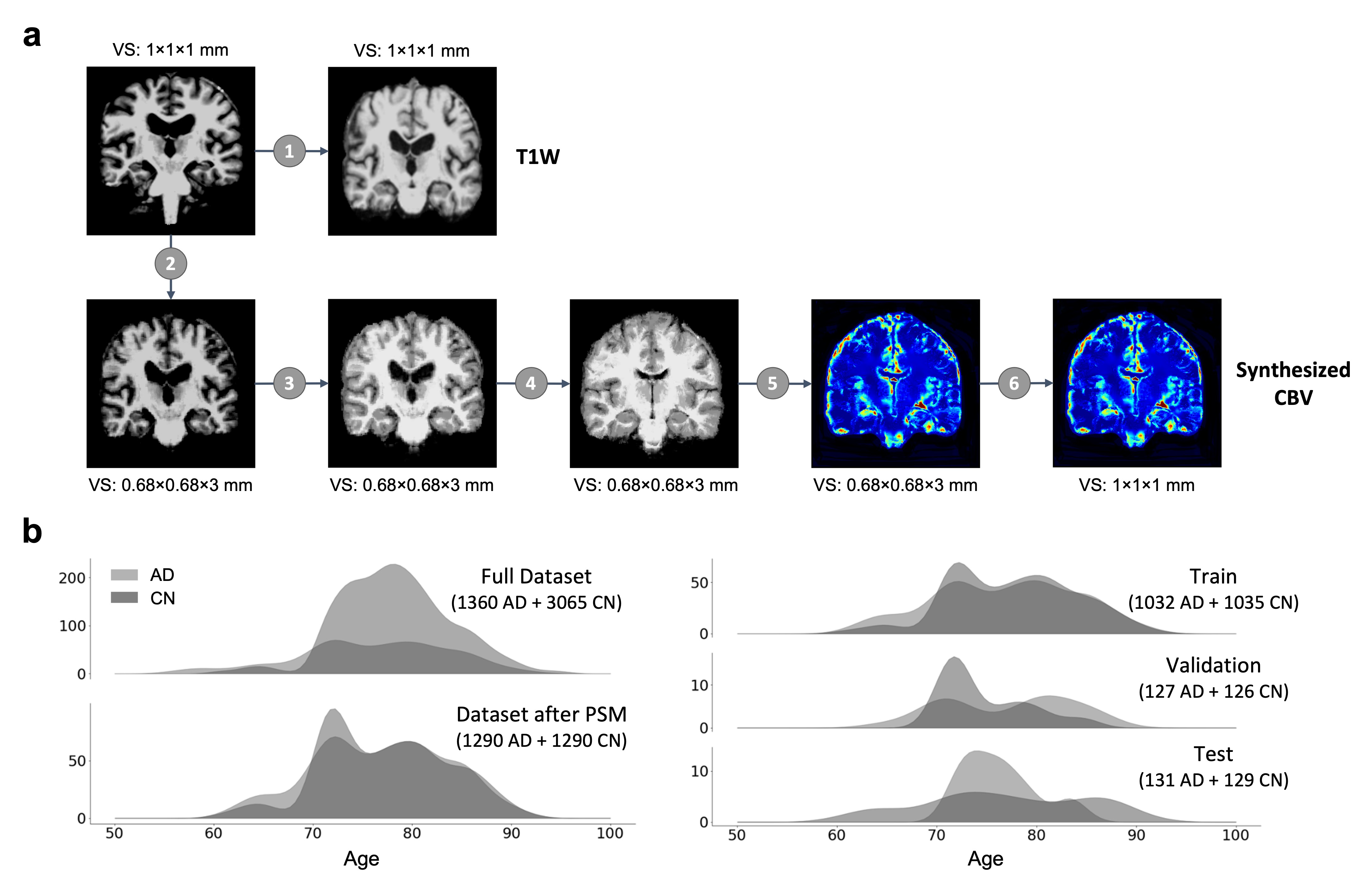

Supplement: Supplementary Figure S4 — Details of MRI processing pipeline and partition of the Alzheimer's Disease (AD) classification dataset. (a) Data processing pipeline to generate the input of different AD classification deep learning models. The preprocessing of structural T1W MR data is necessary to remove unwanted artifacts and transform the data into a standard version before feeding them into the models. For each MRI structural, we process the T1W 3D volume through a standardized pipeline: (1) whole brain affine registration to MNI152 template space and other processing detailed in a prior study (Feng et al., 2018); (2) whole brain rigid registration to MNI152 template space; (3) histogram matching to the DeepContrast CU T1W MRI; (4) whole brain diffeomorphic registration to standard CU template; (5) generating the synthesized CBV maps; (6) up-sampling the synthesized CBV maps to 1-mm isotropic resolution. (b) Left: Age distributions of the participants in the entire dataset (top) and the subset after propensity-score matching of age (bottom). Right: Age distributions of the participants assigned to the train, validation, and test dataset. VS, voxel size; AD, Alzheimer's diseased; CN, cognitive normal. [file Image_4.JPEG]

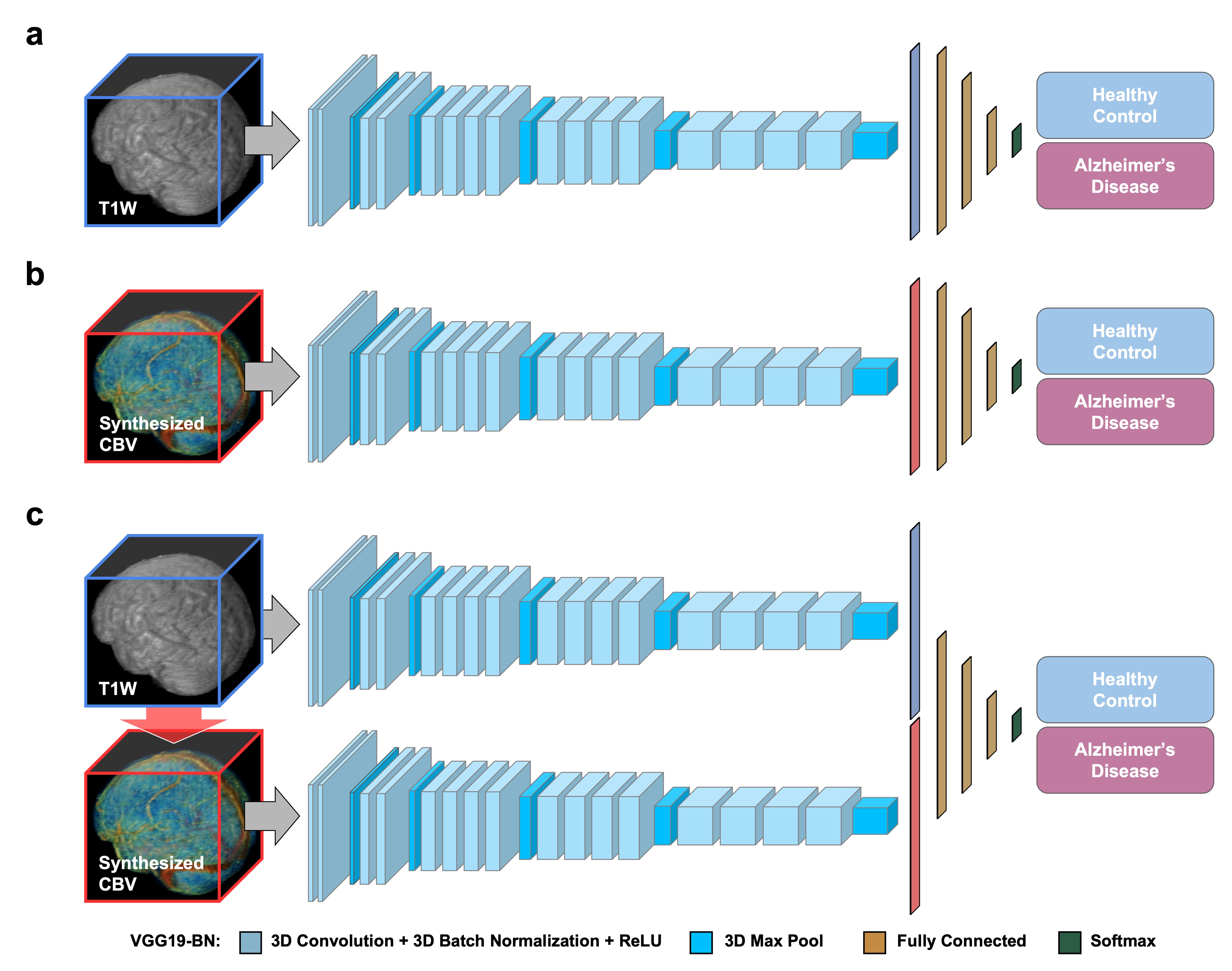

Supplement: Supplementary Figure S5 — Details of the three candidates of AD-classification networks (Networks used in Figure 7). (a) Proposed Model 1. Modified 3D VGG-19 network with batch normalization uses T1W MRI scans as the model input. This model aims to perform the AD classification based on structure T1W MRI scans. (b) Proposed Model 2. Modified 3D VGG-19 network with batch normalization uses synthesized CBV maps as the model input. This model aims to realize the AD classification based on functional MRI maps. (c) Proposed model 3. Modified VGG-19 network with double encoding paths uses T1W MRI scans and the synthesized CBV maps as the model input. Separate encoders in the model have the same structure but different weights. This could help improve the structure and functional information fusion and classification performance. The red arrow represents the generation of synthesized CBV from T1W MRI scans using DeepContrast. [file Image_5.JPEG]
